# Finding the “Dark Matter” in Human and Yeast Protein Network Prediction and Modelling

**DOI:** 10.1371/journal.pcbi.1000945

**Published:** 2010-09-23

**Authors:** Juan A. G. Ranea, Ian Morilla, Jon G. Lees, Adam J. Reid, Corin Yeats, Andrew B. Clegg, Francisca Sanchez-Jimenez, Christine Orengo

**Affiliations:** 1 Research Department of Structural & Molecular Biology, University College London, London, United Kingdom; 2 Department of Molecular Biology and Biochemistry-CIBER de Enfermedades Raras, University of Malaga, Malaga, Spain; University of Chicago, United States of America

## Abstract

Accurate modelling of biological systems requires a deeper and more complete knowledge about the molecular components and their functional associations than we currently have. Traditionally, new knowledge on protein associations generated by experiments has played a central role in systems modelling, in contrast to generally less trusted bio-computational predictions. However, we will not achieve realistic modelling of complex molecular systems if the current experimental designs lead to biased screenings of real protein networks and leave large, functionally important areas poorly characterised. To assess the likelihood of this, we have built comprehensive network models of the yeast and human proteomes by using a meta-statistical integration of diverse computationally predicted protein association datasets. We have compared these predicted networks against combined experimental datasets from seven biological resources at different level of statistical significance. These eukaryotic predicted networks resemble all the topological and noise features of the experimentally inferred networks in both species, and we also show that this observation is not due to random behaviour. In addition, the topology of the predicted networks contains information on true protein associations, beyond the constitutive first order binary predictions. We also observe that most of the reliable predicted protein associations are experimentally uncharacterised in our models, constituting the hidden or “dark matter” of networks by analogy to astronomical systems. Some of this dark matter shows enrichment of particular functions and contains key functional elements of protein networks, such as hubs associated with important functional areas like the regulation of Ras protein signal transduction in human cells. Thus, characterising this large and functionally important dark matter, elusive to established experimental designs, may be crucial for modelling biological systems. In any case, these predictions provide a valuable guide to these experimentally elusive regions.

## Author Summary

To model accurate protein networks we need to extend our knowledge of protein associations in molecular systems much further. Biologists believe that high-throughput experiments will fill the gaps in our knowledge. However, if these approaches perform biased screenings, leaving important areas poorly characterized, success in modelling protein networks will require additional approaches to explore these ‘dark’ areas. We assess the value of integrating bio-computational approaches to build accurate and comprehensive network models for human and yeast proteomes and compare these models with models derived by combining multiple experimental datasets. We show that the predicted networks resemble the topological and error features of the experimental networks, and contain information on true protein associations within and beyond their constitutive first order binary predictions. We suggest that the majority of predicted network space is dark matter containing important functional areas, elusive to current experimental designs. Until novel experimental designs emerge as effective tools to screen these hidden regions, computational predictions will be a valuable approach for exploring them.

## Introduction

Many features of biological systems cannot be inferred from a simple sum of their components but rather emerge as network properties [1]. Organisms comprise systems of highly integrated networks or ‘accelerating networks’ [2] in which all components (proteins, lipids, minerals, water, etc.) are integrated and coordinated in time and space. Given such complexity, the gaps in our current knowledge prevent us from modelling complete living organisms [3], [4]. Therefore, the development of bio-computational approaches for identifying new protein functions and protein-protein functional associations can play an important role in systems biology [5].

The scarce knowledge of biological systems is further compounded by experimental error. It is common for different high-throughput experimental approaches, applied to the same biological system, to yield different outcomes, resulting in protein networks with different topological and biological properties [4]. However, errors are not restricted to high-throughput analysis. For example, it has been demonstrated that high-throughput yeast two-hybrid (HT-Y2H) interactions for human proteins are more precise than literature-curated interactions supported by a single publication [6].

There has been a great deal of work analysing biological networks across different species, giving insights into how networks evolve. However, many of these publications have yielded disparate and sometimes contradictory conclusions. Observation of poor overlap in protein networks across species [7] and divergence amongst organisms [8] suggest fast evolution. Significant variation in subunit compositions of the functional modules has also been observed in protein networks across species [9]. However, in contrast to these observations, recent work using combined protein-protein interaction data suggests high conservation of the protein networks between yeast and human [10]. This approach, based on data combination, stresses the importance of integrating different data sources to reduce the bias associated with errors in functional prediction, and to increase the coverage in network modelling, and has been demonstrated in numerous studies [11]–[14].

Increasing the accuracy of networks by integrating different protein interaction data relies on the intuitive principle that combining multiple independent sources of evidence gives greater confidence than a single source. For any genome wide computational analyses, we expect the prediction errors to be randomly distributed amongst a large sample of true negative interactions (i.e. the universe of protein-protein interactions that do not take place). Hence, it is unlikely that two independent prediction methods will both identify the same false positive data in large interactomes like yeast or human. In general, we expect the precision to increase proportionally to the number of independent approaches supporting the same evidence.

From the available list of well-known integration methods specifically designed to integrate diverse protein-protein interaction -PPI- datasets (e.g. Naïve-Bayes; SVM; etc. [15]–[19]), we chose the Fisher method [12] in order to have a predictor that is independent from the experimental data used to validate it. Fisher integration method is not a trained or supervised method as, for example, Naive Bayes or SVM methods. The Fisher method presumes a Gaussian random distribution of the prediction datasets' scores as a null hypothesis and the Fisher integrated score calculation is based on Information Theory statistics [20], [21]. Therefore, the Fisher integration score is completely independent of the experimental datasets used in this study to validate and compare the predictions.

In this work, we significantly increase the prediction power of binary protein functional associations in yeast and human proteomes by integrating different individual prediction methods using the Fisher integration method. Three different untrained methods are implemented: GECO (Gene Expression COmparison); hiPPI (homology inherited Protein-Protein Interactions); and CODA (Co-Occurrence Domain Analysis) run with two protein domain classifications, CATH [22] and PFAM [23] (see the section: *Ab initio* methods used for building the Predictograms). The four different prediction datasets obtained by these methods (GECO, hiPPI, CODAcath and CODApfam), were integrated using simple integration and Fisher's method as examples of untrained methods (see the section: Integrating the prediction data). Similarly *ab-initio* prediction datasets from STRING [14] were also integrated using Fisher integration and compared against the integrated prediction datasets from our methods. Results from the Fisher integration of our prediction datasets were benchmarked and compared against the individual prediction methods and the results from the integrated STRING methods. In all cases we demonstrate increased performance for the integrated approach (assessed by prediction power) with the Fisher integration of GECO, hiPPI, CODAcath and CODApfam datasets yielding the best results.

Protein pairs identified by significant Fisher integration p-values were used to build a protein network model for yeast and human proteomes referred to as the Predictogram (PG). Additionally, all the protein-protein associations from several major biological databases, including Reactome [24], Kegg [25], GO [26], FunCat [27], Intact [28], MINT [29] and HRPD [30] were retrieved and combined into a network referred to as a Knowledgegram (KG). As implemented in other pioneering studies [31], we built predicted (PG) and experimental (KG) models for further comparison. Different network topology parameters were calculated and compared between KG and PG models for two test species *Homo sapiens* (human) and *Sacharomyces cerevisae* (yeast). We observe how the networks change as the cut-off on the confidence score of the predictions is varied. Results of this PG and KG network comparison demonstrate that PG networks resemble KG networks in many of the major topological features and model a substantial fraction of real protein network associations, as previously observed in some bacterial predicted networks [32], [33].

There have been frequent observations of low overlaps between different experimental high-throughput approaches [34]. Our comparison of the PG and KG models also show that the intersection between the two models is small and that the majority of predictions in the PG are “novel predictions”. However, the overlap between PG and KG is significantly higher than expected by random in both species supporting a correspondence between the PG and KG screenings of PPI space. This PG and KG data overlap is significantly larger in yeast than in human, pointing to a better functional characterization of the yeast PPI network and the presence of larger dark areas in the human PPI network still hidden from current experimental knowledge. We suggest that this novel prediction set may be a valuable estimation of the relative differences in “dark matter” of uncharacterised protein-protein associations between both specie, and we show that this dark matter contains key elements, such as hubs, with important functional roles in the cell.

By analogy [35], “dark matter” in protein network models refers to predicted protein-protein associations, whose existence has not yet been experimentally verified. In this study, we suggest that dark matter involves functional associations difficult to characterise by current experimental assays making any network modelling of organisms highly incomplete and therefore inaccurate.

The results are divided into four main sections in which the predicted and experimental PPI models of human and yeast are compared. The first section analyses the performance of the single and integrated methods predicting the protein associations and determines the correlation between the prediction scores and the degree of accuracy and noise in the predictions. The second chapter compares the topological network features of the predicted and experimental PPI models at equivalent levels of accuracy and noise. The third section searches for functional differences between the predicted and experimental models looking for specific functional areas which appear to be illuminated by the prediction methods but elusive to the experimental approaches. Whilst the final fourth section explores whether the predicted PPI network graphs contain additional context-based information on protein associations beyond the sets of predicted protein pairs used to build the networks.

## Results

### PG model integration, benchmarking, and analysis

The different methods for predicting protein-protein interactions and functional associations were run on the whole yeast and human proteomes, generating four prediction datasets for each organism, GECO, CODAcath, CODApfam and hiPPI (see the section: Running the PG methods on the human and yeast proteomes and section 1 in Text S1). Each of these methods produces an untrained score value, which was normalized to a p-value, reflecting the reliability of the predictions (see the section: P-value calculation).

**Figure 1:**
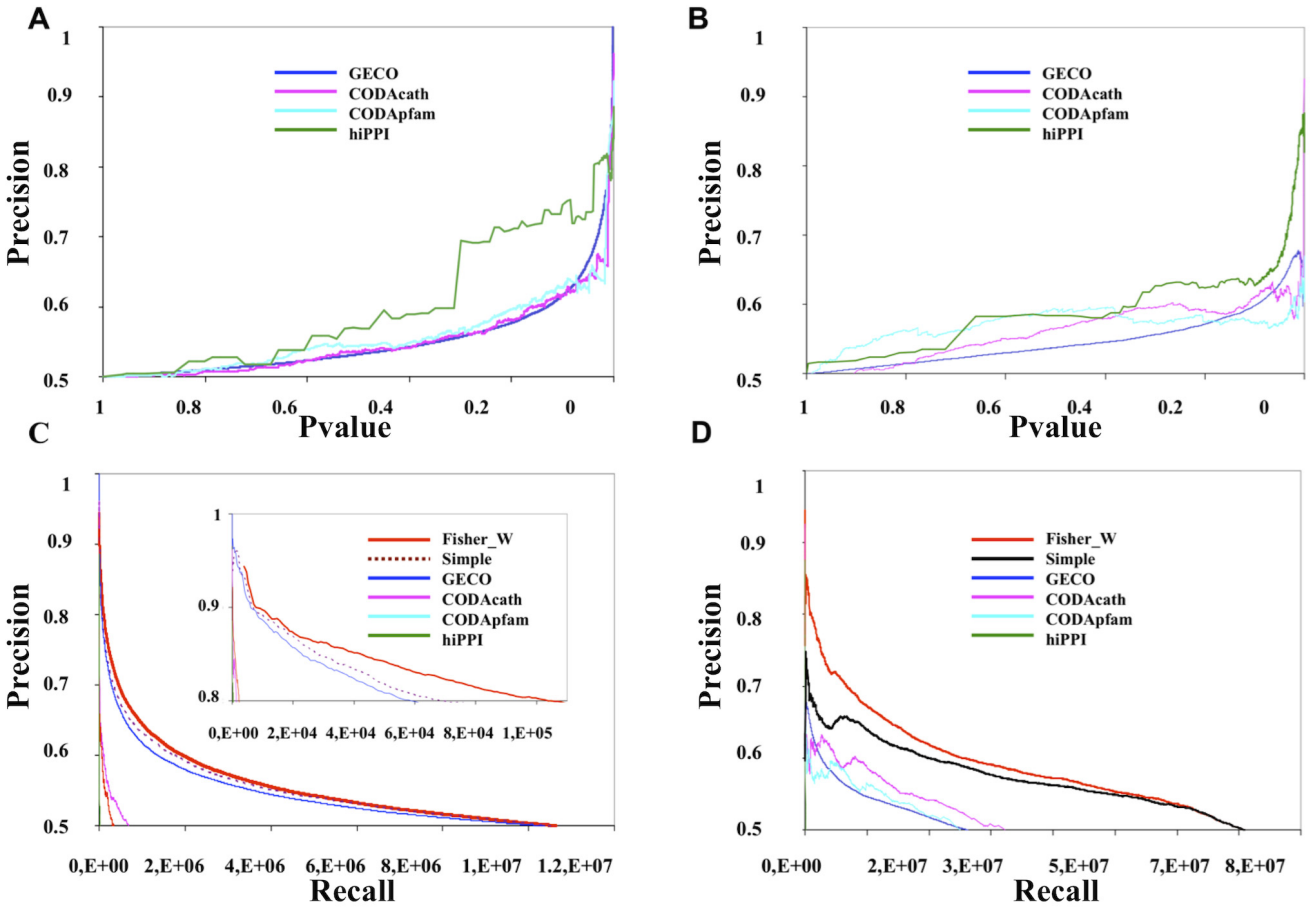
Results of the benchmark studies for the individual prediction methods and the integrated methods. A and C plots are from Yeast datasets and B and D are for Human results. A and B plots show precision versus p-values and C and D graphs show precision versus recall. Inset to the C plot shows an enlargement to visualize the improvements obtained by using the Fisher integration in yeast.

Benchmark datasets for each organism, comprising reliable protein pairs based on Gene Ontology Semantic Similarity scores (referred to as the Goss refined – Gossr datasets; see the section: The GO Semantic Similarity refined dataset (Gossr) used for validating the prediction methods), were used to assess performance (note that the performance measured will depend on the quality of the validation dataset; see section 2 in Text S1; [4]). Precision values are estimated by comparing the methods performance in predicting true PPI versus a random predictor, used to calculate the FP (False Positive) rates (see the section: Precision and Recall calculation). We find that for all methods the p-values correlate inversely with the precision scores, in both proteomes, as expected if genuine functional information is linked to the prediction score (Figures 1a and b). It is possible that a randomly selected PPI could be a TP by chance. However, this is likely to be a rare event and in any case it will mean that we tend to underestimate the performance of the methods as it would mean we are overestimating FPs, from our random model (see section 2 in Text S1).

The mutual information scores demonstrate the independence of the 4 different prediction datasets (see section 3 in Text S1). The p-values from the 4 prediction datasets were integrated using Fisher and Simple integration, both of which are untrained integration methods (see the section: Integrating the prediction data).

Precision (TP/TP+FP) versus Recall (Recall considered as the number of predicted hits) is plotted for yeast and human Gossr validation (Figures 1c and d), for all the individual and integrated methods in order to compare their statistical prediction power (prediction power equals the area under the Precision vs. Recall curves). The prediction powers of all of the integrated methods outperform any individual method. Increase of the prediction power following integration is especially pronounced in human. Whilst less pronounced, the increase in yeast remains significant above 80% precision (zoom over Figure 1c). At these higher precision levels differences in the predictive powers become very significant with the Fisher integration methods approximately doubling the recall for a given precision over the best single or simple integration methods (around 90,000 predictions with Fisher compared to around 60,000 predictions with simple integration, see the abscissas axis in zoom of Figure 1c).

We have performed additional validation of the Fisher method using a set of physical interacting pairs as gold standards in yeast and human (see section 4 in Text S1). Validation with the Int (physical interaction) dataset in yeast (Figure S3a in Text S1) assigns a higher precision ≥90% to the same predicted dataset of around 90,000 top ranked Fisher predictions, which were calculated with a precision ≥80% in the Gossr validation (Figure 1a). Whilst in human the Int and Reactome_int (physical interaction) validations (Figure S3b in Text S1) yielded precisions of ≥76% and ≥82% for the same top ranked Fisher dataset that was assigned a precision ≥80% in the Gossr validation (Figure 1b). All these validations indicate that Fisher p-values scores are also linked to physical protein-protein interactions with a similar consistent reliability of around 80% precision as shown in the Gossr validation.

Fisher was also implemented to integrate similar datasets of individual STRING *ab-initio* predictions (gene neighbourhood, co-occurrence, fusion, and co-expression) in yeast and human. FisherW integration of the STRING datasets showed a significantly lower performance compared to the GECO, CODAcath, CODApfam and hiPPI Fisher integration (see section 5 in Text S1). Using Gossr as the training dataset, GECO, CODAcath, CODApfam and hiPPI prediction datasets were also integrated by Bayes (see section 6 in Text S1). Bayes integration produced uneven results in yeast and human compared to Fisher, whilst Fisher outperforms Bayes for the highest levels of precision in yeast (see left side of the Figure S5a in Text S1), in human Bayes performs better (see Figure S5b in Text S1). From these results we observed that the Fisher integration yields a good performance compared to using a trained method (i.e. Bayes), despite the fact that the latter has benefit of learning from the experimental (KG) information to predict PPIs.

In all cases (yeast and human) Fisher integration of the GECO, CODAcath, CODApfam and hiPPI predictions was shown to be a powerful combination which significantly increases the prediction power without using any KG trained or supervised algorithms. This premise is crucial if we aim to detect genuine similarities between the PG and KG models, unbiased by overlap between supervised predictions and their training sets (as would occur by using a Bayes integration). Because of this the Fisher weighted predictions were chosen for generating the PG network models used in subsequent analyses of the networks.

### Comparison of the topological features of the KG and PG networks

To test whether the PG networks based on the binary predictions share features with networks built on reliable KG evidences, different topological parameters (see section 7 in Text S1) were calculated and compared between PG and KG networks. This analysis was carried out at different levels of significance in the yeast and human proteomes.

Different PG networks were constructed from the binary predictions by varying the link (edge) p-value cut-off. This was done for a range of p-values from p-value≤0.001 (PG_0.001_) to p-value≤1.0 (PG_1.0_). KG network models were also tested at different levels of confidence based on the number of KG evidences supporting the same protein-protein associations. Mutual information calculation on the KG data showed broad independence except for the Goss and Foss (FunCat semantic similarity) datasets, therefore Goss and Foss evidences were summed and considered as a single dataset of KG evidences. Different KG networks were constructed by varying the minimum number of independent evidences required to form an edge/link. Random models were also generated for all the PG and KG networks as described in the section: Network randomisation. The PG, KG and their corresponding randomised networks, built at different significance levels, provide comparable frameworks for examining the topological properties of biological networks.

**Figure 2:**
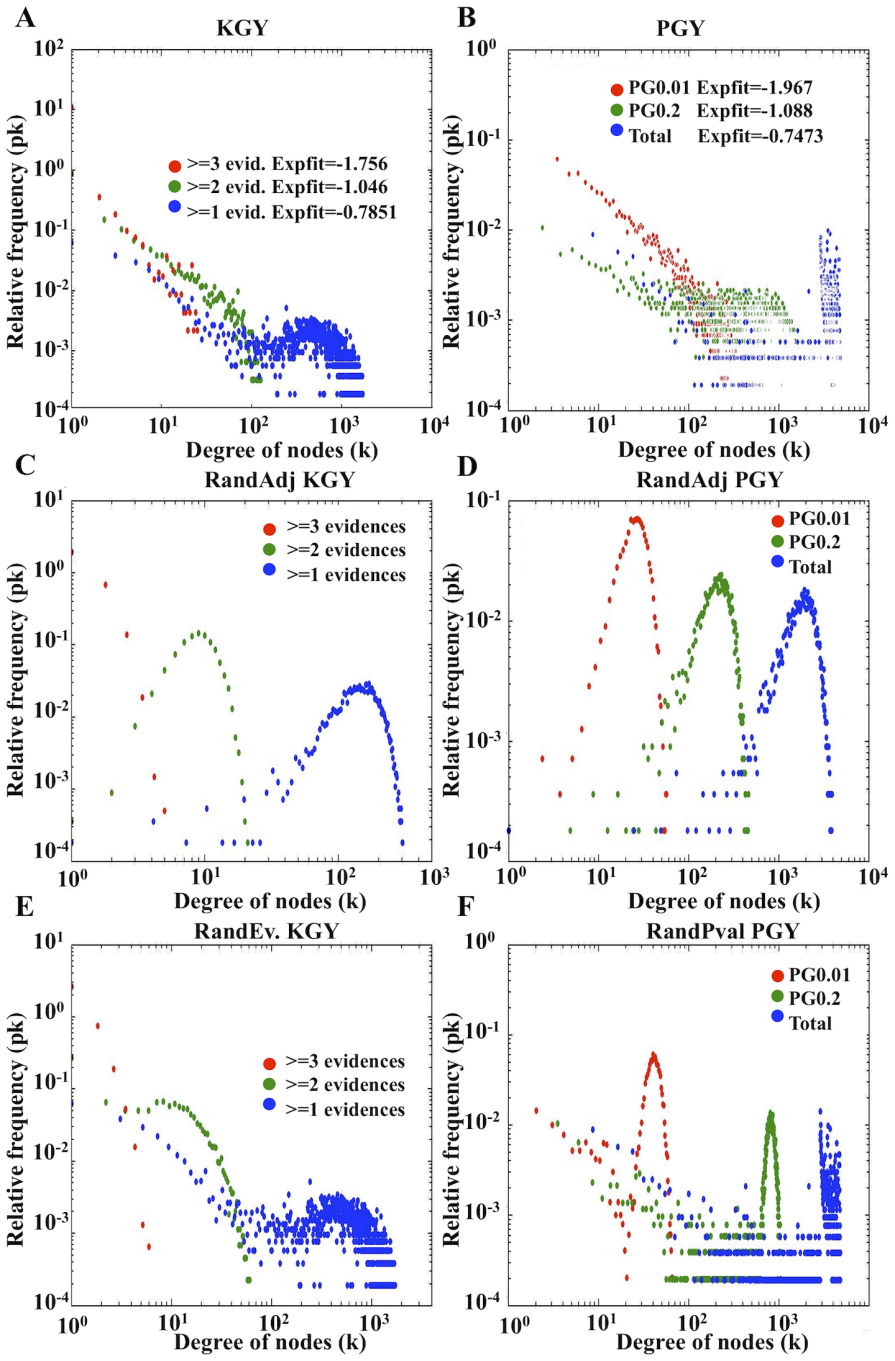
Yeast degree distribution for the various networks analysed. Panels A and B correspond to the KG and PG networks respectively, the legend for these panels show the correlation coefficients and exponents corresponding to the linear regression fit of the data. The corresponding randomised networks are shown below for KG (panels C, E) and PG (panels D, F) networks respectively. Panels C and D are from network randomisations by the adjacency method (see the section: Network randomisation). Panels E and F randomisations are from the evidence and p-value shuffling respectively.

**Figure 3:**
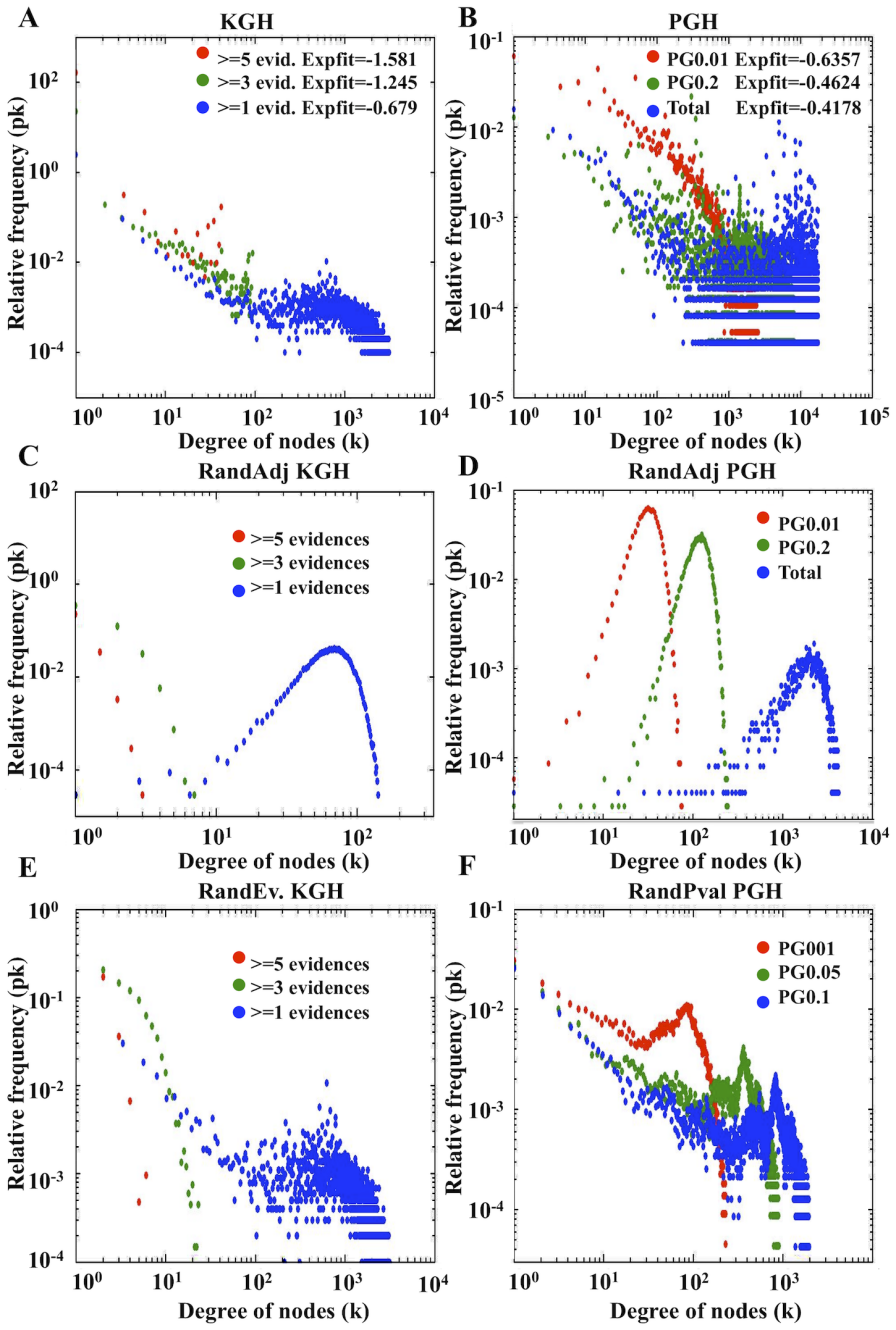
Human degree distribution for the various networks analysed. Panels A and B correspond to the KG and PG networks respectively, the legend for these panels show the correlation coefficients and exponents corresponding to the linear regression fit of the data. The corresponding randomised networks are shown below for KG (panels C, E) and PG (panels D, F) networks respectively. Panels C and D are from network randomisations by the adjacency method (see the section: Network randomisation). Panels E and F randomisations are from the evidence and p-value shuffling respectively.

Real biological networks have been shown to have a scale-free topology with a high degree of clustering [36]. Scale free networks show, amongst other characteristics, a power law distribution in the frequencies of connectivity of their nodes (k_i_) with values for the exponent between 2 and 3 [37], [38]. When the frequency distribution for node connectivity (k_i_) is plotted for the PG and KG networks, constructed at different confidence levels for yeast and human, we observe a trend towards higher exponents in the fitted power law functions as the network reliability increases (Figures 2a, 2b, 3a and 3b; and Figure S6a–Figure S8a in Text S1). The trend toward scale-free organisation is more significant in yeast than in human KG and PG models, with exponent values that get close to 2 for the most reliable network levels (see PG_0.01_ and KG_≥3 evid._ distributions in Figures 2a and b), whilst in human KG and PG models the exponents are systematically lower than in yeast, at equivalent levels of significance (Figures 3a and b).

Yeast and human KG and PG models show non-random distributions of their degree (k_i_) frequencies for all levels of network reliability tested, except for the lowest level (Figures 2 and 3, compare plots a and b, c and d, e and f; and Figure S6, Figure S7 and Figure S8 in Text S1 compare plots a, b and c). In contrast to the real PG and KG models the *adjacency randomised networks* in yeast and human show a Gaussian distribution with node degree (Figures 2 and 3, plots c and d versus a and b). A Gaussian distribution is also observed in the *p-value random models* but for high node degree only (Figures 2 and 3, plots e and f). A Gaussian distribution, typical of random behaviour, is also observed for the KG and PG networks built at the lowest level of statistical reliability (compare PG_total_ and KG_total_ in Figures 2 and 3, plots a, b, e and f). However, this Gaussian random distribution disappears when edges with weaker statistical weight are removed in increasingly more significant PG and KG networks, indicating the correlation between edge statistical weight (p-values and # of evidences) and the non-random scale free topology expected in real biological networks.

Power-law degree distribution is a necessary but not a sufficient characteristic of scale free networks. Therefore, other topological features of the KG and PG networks were measured in order to give more support to the hypothesis of scale free tendency for our models. These included: average clustering coefficient; assortativity; or network hierarchy amongst other parameters described in the section: Network topology structure characterisation and the section 7 in Text S1.

The trend of increasing average clustering coefficient with increasing network reliability (KG and PG network models built at more highly significant p-values and # evidences levels) lends further support to the scale-free organization of the KG and PG networks in yeast and human (see Figure S6d–Figure S8d in Text S1). Node assortativity (or preferential attachment of the nodes) is another topological parameter that supports the scale-free trend of the KG and PG models in yeast and human, (see section 11 in Text S1, and Figure S9 and Figure S10 in Text S1; [36]). The assortativity observed in KG and PG models indicates a network organization close to a real network in stark contrast to the random models [39], [40].

Network hierarchy is another topological feature that can be considered by using the logarithmic distribution of the clustering parameter [41]. For all our KG and PG networks we observed a flat distribution (no correlation between clustering coefficient and connectivity –k_i_-) implying a non-hierarchical organization, since hierarchical organization exhibits a power-law distribution of these two parameters (see section 12 and Figure S11 in Text S1). This result, taken together with the observations on clustering distribution, indicates a modular organisation of the KG and PG networks [41]. This would explain why these networks tend towards, but never completely reach a scale-free distribution exponent [36]. The modularity of the KG and PG networks (see section 13 and Figure S12 in Text S1) is also supported by other conventional network parameter values measured for these networks and presented in Table S3 and Table S4 in , such as: network density; cluster average (triangle formation likelihood); characteristic path length; network radius; and diameter. Radius and diameter are only measured for the largest connected component of the network [40], [42].

### Analysing the ‘dark matter’ in the PG models

KG models represent the known (experimentally determined) protein associations while PG models represent sets of associations predicted by *ab-initio* methods. We wanted to estimate the extent of ‘dark matter’ in the yeast and human networks by comparing how much of the predicted network space was not covered by experimental evidence in both specie. We also investigated the presence of hubs in the PG dark matter and the functional characteristics of these dark (hidden) hubs.

We used the most reliable (precision≥80%) PG models (PG_0.01_ in yeast – about 90,000 pairs - and PG_0.014_ in human – about 10^6^ pairs; see section 15 and Table S5 in Text S1) to estimate the intersection with the KG models for the two organisms (Table 1). In yeast, the percentage of edges (18%) overlapping between the KG and PG models is larger than for the human models (1.34%; Table 1). That is, 18% of the predicted protein-protein associations in yeast PG_0.01_ model are backed by experimental evidences in the KG set, which is a highly significant figure compared to any of the random models (18.22/1.34 = 13.60 times higher than R.1 model, and 18.22/4.26 = 4.28 times higher than R.2 model; see Table 1).

**Table 1:** PG and KG networks intersection analysis.

	Type	Networks	# Edges	# Nodes	#Ed./#Nod.	%PGe
**Yeast**	**Real**	KG/PG_0.01_	17,373	2,293	7.6	18.22
	**R. 1**	KG/PG_0.01_	1,280	2,707	0.5	1.34
	**R. 2**	KG/PG_0.01_	4,062	4,279	0.9	4.26
**Human**	**Real**	KG/PG_0.014_	14,048	3,958	3.5	1.34
	**R. 1**	KG/PG_0.014_	898	1,111	0.8	0.08
	**R. 2**	KG/PG_0.014_	3,073	5,633	0.5	0.29

From left to right: the *yeast* and *human* data division; *Type*, real data or random model; *Networks*, intersections of network models; *# Edges*, number of protein pairs in the intersections; *# Nodes*, number of different proteins (nodes) in the intersections; *#Ed./#Nod.*, or network density is ratio of the number of edges divided by the number of nodes; *%PGe*, percentage of the PG model's edges backed by the KG model. *R. 1*, *p-values random model* and *R. 2*, *adjacency random model* (see the section: Network randomisation) the randomisation process was realized over the matrix of possible binary associations of all the proteins (nodes) in the PG and KG models. Calculation of intersections for the random models went through 1,000 iterations. For further statistics see Table S2 in Text S1.

The percentage of edge predictions, backed by experiments, drops considerably for human. Only 1.4 percent of the predicted protein associations (PG) were also present in the KG model (Table 1). Although, the percentage of experimentally backed predictions (1.34%) is significantly higher than expected by random (1.34/0.08 = 16.75 times higher than R.1 model, and 1.34/0.29 = 4,62 times higher than R.2 model; see Table 1).

The density of the overlap between PG and KG in yeast (#Ed./#Nod. = 7.6 in Table 1) is double the human value (3.5), and in both cases is considerably more than the expected random density (Table 1). Additionally, the percentage of proteins (nodes) in the PG model without known experimental association in the KG model is about 30 times smaller in yeast (105 nodes/4374 nodes  = >2.4% see Table S5 in Text S1) compared to the human PG model (13,961 nodes/19,618 nodes  = >71.2% see Table S5 in Text S1).

These statistical analyses of the PG and KG intersections indicate that about 82%, in yeast, and 98% of predicted protein associations in human are not backed by experimental evidence in the KG model, giving an estimate of dark matter in the yeast and human protein networks. Only 2.4% of the PG proteins in yeast are dark nodes (proteins without experimental association in the KG model), whilst dark nodes constitute 71% in human.

Although PG and KG models explore significantly different regions of protein binary association space, interestingly, given the small intersections and density values, the PG and KG overlap is still significantly larger than expected by random (Table 1), indicating the overall coherence of the PG and KG models despite the presumably huge size of real protein network space. It is likely that protein network space is much larger in human than in yeast, given their respective proteome sizes, which presumably explains the higher proportion of dark matter in the human compared to the yeast PG networks.

Enrichment of the degree of a node in the PG model (PGk_i__er) was calculated in order to measure the difference in the connectivity (k_i_) values for a protein in the PG and KG networks (see the section: Calculating the PGk_i_ enrichment ratio and the PG functional enrichment). A high PGk_i__er value indicates the presence of a dark (experimentally hidden) hub, a protein with many predicted associated proteins in the PG model and few, if any, experimentally validated KG associations. Proteins in the yeast and human PG models were ranked using their PGk_i__er value, retrieving the top 10 ranked proteins for both organisms (see Table 2) as the most likely representatives of predicted dark hubs.

**Table 2:** Ten top proteins in the yeast and human PGk_i__er ranked lists.

Yeast Prot. Acc. N.	KG k_i_	PG k_i_	PG k_i__er	R.	Gene name	Uniprot descriptions
Q07928	0	213	213	1	GAT3	GATA-type zinc finger: transcription factor activity (Inferred from electronic annotation). Unknown function.
P47055	0	201	201	2	LOH1	Multi-pass membrane protein. Possibly involved in maintaining genome integrity
A6ZR40	0	189	189	3	SCY_1587	Predicted protein, unknown function.
Q12079	0	188	188	4	YPR027C	Multi-pass membrane protein. Uncharacterized membrane protein YPR027C
P53964	0	187	187	5	YNL033W	Single-pass membrane protein. Uncharacterized membrane protein YNL033W
P47056	0	172	172	6	YJL037W	Multi-pass membrane protein. Uncharacterized protein YJL037W
P09937	0	171	171	7	SPS4	Sporulation-specific protein 4. Not essential for sporulation. Might be a component of the cell wall.
P32643	0	163	163	8	TMT1	Trans-aconitate 3-methyltransferase. Inducted during amino acid starvation.
A6ZV06	0	155	155	9	SCY_2239	Predicted protein with alpha/beta hydrolase fold, unknown function.
A6ZP11	0	151	151	10	SCY_5229	Predicted protein with a Nucleotide binding domain potentially found in RNases, unknown function.

Human Prot. Acc. N.	KG k_i_	PG k_i_	PG k_i__er	R.	Gene name	Uniprot descriptions
Q6ZP81	0	2597	2597	1	-	Highly similar to Homo sapiens titin (TTN), with Fibronectin type III domain. Unknown function.
Q9UM08	0	2214	2214	2	HGC6.3	Unknown function
Q9BZ69	0	1860	1860	3	P143	Predicted membrane protein with histidine kinase domain, two-component sensor activity (Inferred from electronic annotation). Unknown function.
Q0VAC6	0	1828	1828	4	SUMF1	Unknown function
Q5JRQ2	0	1796	1796	5	NT5E	5′-nucleotidase, ecto (CD73). Hydrolase activity; nucleotide catabolic process.
Q96F04	0	1759	1759	6	MMP28	Matrix metallopeptidase 28. Predicted protein with a putative peptidoglycan binding domain.
Q3KR05	0	1702	1702	7	NEU4	Sialidase 4. Unknown function.
Q32MK0	0	1660	1660	8	MYLK3	Putative myosin light chain kinase 3. May play a role in smooth muscle contraction.
Q4ZG20	0	1657	1657	9	TTN	Putative uncharacterized protein TTN. Unknown function.
Q96CP1	0	1602	1602	10	RELA	Predicted protein with a transcription factor Rel homology domain (RHD)

From left to right: ***Prot. Acc. N.***, Protein accession number in Uniprot database; ***KGk_i_***, protein connection degree (k_i_) in the KG network; ***PGk_i_***, protein ki in the PG network; ***PGk_i__er***, protein k_i_ enrichment ratio in PG compared to KG network; ***R.***, rank in the PGk_i__er list; ***Gene name*** in Uniprot and ***Uniprot*** functional ***description***.

A common interesting feature of dark hubs, shown in Table 2, is that almost all of them correspond to predicted proteins with only electronically inferred or unknown functions in Uniprot. This is expected for proteins which are absent from the KG model and therefore have no associated functional evidence. This overrepresentation of functionally unknown proteins in the set of dark hubs is also supported by extensive functional annotation searches using the DAVID algorithm [43] in yeast and human (see section 16 in Text S1). Although enrichment in predicted datasets of uncharacterised proteins has also been observed in earlier studies by other groups [31], it was not used to identify sets of dark hub proteins, as in our study. Here, we identify highly connected and therefore topologically important nodes in the PPI networks currently lacking direct experimental information.

We analysed the top 10 dark hubs in the yeast PG network using functional annotation inferred by homology, these proteins correspond mainly to membrane embedded proteins, although there are also proteins related to other disparate functions, such as: transcription factors, RNase (probably involve in siRNA degradation processes), sporulation, and various enzymes (see Table 2). Enrichment bias in “integral to membrane proteins” is statistically significant in the yeast dark hubs dataset comparing the extremes of the PGk_i__er ranked list with the DAVID algorithm (see section 16 in Text S1). Functions for the top 10 dark hubs in humans are even broader than in yeast including proteins with Fibronectin domains, kinases with presumably sensor or motor functions, an Ecto-5′-nucleotidase probably involved in extracellular nucleotide catabolism [44], a transcription factor, and a matrix metallopeptidase amongst other proteins of completely unknown function (see Table 2).

In order to study possible bias in the functional niches highlighted by the PG predictions but absent in the KGs, functional enrichment in the yeast and human PGk_i__er ranked lists was estimated using the GOrilla server [45] and the annotations of the respective proteomes in the GO database examined (see Table 3). Functional enrichment at the top of the ranked lists implies the existence of dark functional niches which are more accessible to *ab-initio* predictions than to experiments.

**Table 3:** Human PGk_i__er ranked list enrichment analysis in the GO database.

Biological process GO term name	GO code	P-value	N	B	n	b	E.
Protein amino acid phosphorylation	GO:0006468	7.63E-22	12769	508	991	111	3
Regulation of small GTPase mediated signal transduction	GO:0051056	1.64E-13	12769	124	982	39	4
>Regulation of Ras protein signal transduction	GO:0046578	2.27E-10	12769	85	982	28	4

Molecular function GO term name	GO code	P-value	N	B	n	b	E.
Protein kinase activity	GO:0004672	3.62E-21	12769	480	991	10	3
>Protein serine/threonine kinase activity	GO:0004674	4.83E-14	12769	349	984	75	3
>Protein tyrosine kinase activity	GO:0004713	2.73E-10	12769	146	972	37	3
GTPase regulator activity	GO:0030695	7.06E-13	12769	319	908	63	3
>Guanyl-nucleotide exchange factor activity	GO:0005085	1.92E-11	12769	123	982	36	4
>Small GTPase regulator activity	GO:0005083	1.59E-10	12769	211	982	47	3
ATP binding	GO:0005524	4.61E-25	12769	1097	991	190	2

For the ***Biological processes*** and ***Molecular functions*** GO categories, from left to right: ***GO term name***, name of the enriched GO term; ***GO code***, the term's code in the GO database; ***P-value***, is the enrichment p-value computed according to the GOrilla server [45]; ***N***, is the total number of genes in the ranked list; ***B***, is the total number of genes associated with the specific GO term in the whole ranked list; ***n***, is the total number of genes in the selected top of the list; ***b***, is the number of genes in the selected top of the list associated with the specific GO term; ***E.***, Enrichment (N, B, n, b) = (b/n)/(B/N). Parent-child relationships between GO terms are indicated with the “>” symbol.

GOrilla did not find any significant functional enrichment bias (P-value>E-9) in the yeast ranked list, but detected enrichment of some GO terms in the human ranked list associated with particular biological processes and molecular function categories in GO (see Table 3). Dark (or experimentally hidden) functional niches in the human PG models correspond to key biological processes such as kinase driven regulation through protein amino acid phosphorylation and the regulation of GTPase mediated signalling, including the regulation of Ras protein signal transduction. The ATP binding GO molecular function enrichment is mainly associated with enrichment of kinases.

### Functional association predictions based on context information in the PG networks

If the reliable PG_0.01&0.014_ pairwise predictions capture a significant percentage of true functional relationships and the PG_0.01&0.014_ networks show most of the topological properties of KG networks, it is reasonable to expect that the topology associations in these PG_0.01&0.014_ networks will resemble real biological networks. In other words, we should be able to exploit information on the context of a protein (i.e. connections in the network) to predict associations it has with other proteins sharing a similar context.

In order to test this hypothesis, functional predictions were generated for additional protein pairs, by comparing the interactions of the respective proteins in these pairs, in the PG networks. The results were then validated using the gold standard KG protein pairs' datasets.

This context analysis of the PG networks [32], which involves making predictions based on predictions, is what Mathematical Logic terms a second order analysis. The PG_0.001&0.0014_ pair-wise predictions' datasets used to build the networks in first place are considered the first order predictions in this work (see section 17 in Text S1).

**Figure 4:**
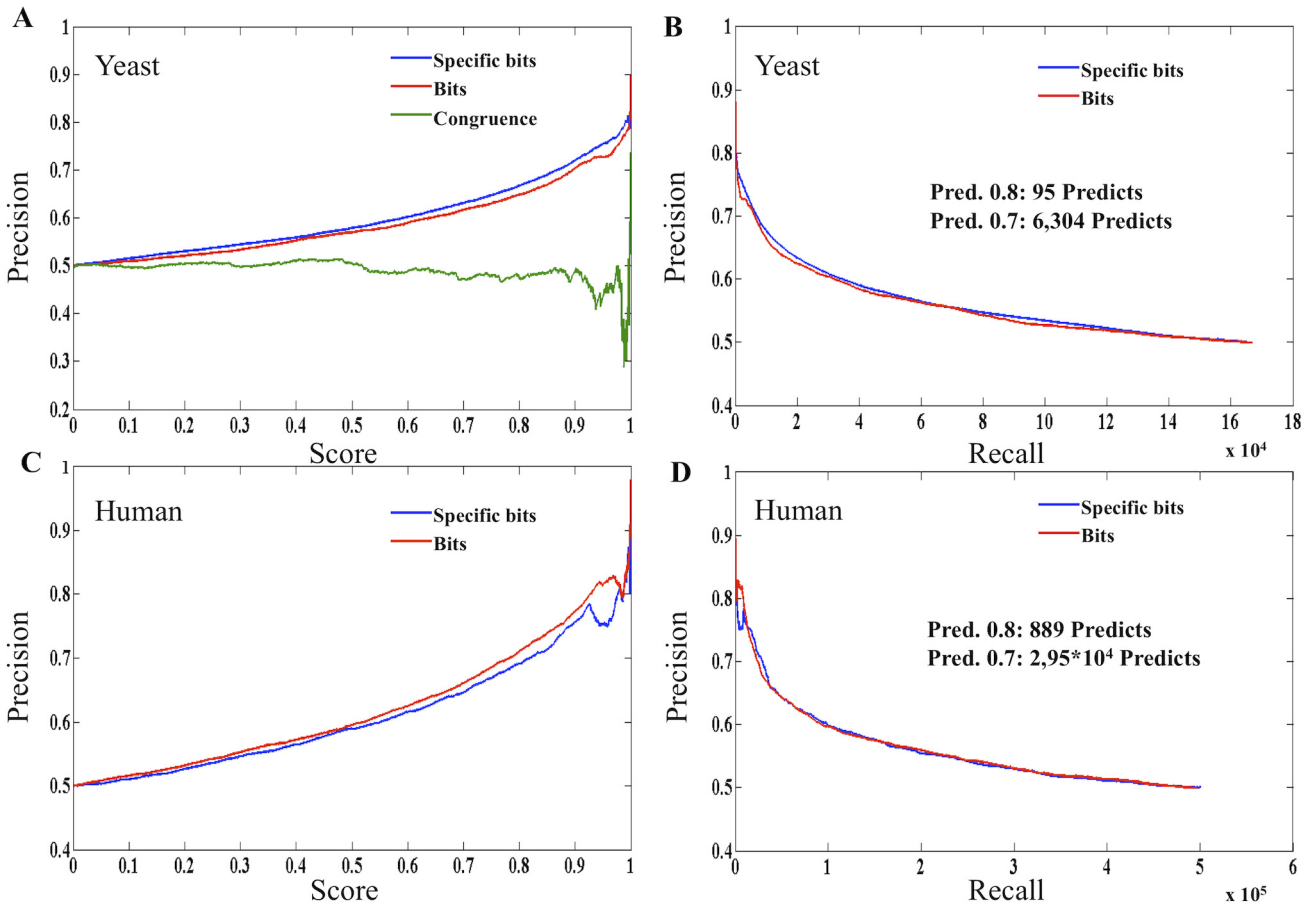
PG networks functional context validation by the KG≥2 evidences dataset. These plots present the precision value (y-axis) versus specific bits similarity score between the interaction profiles of the protein pairs (x-axis in plots A and C) and versus Recall (# of pairs predicted, x-axis in plots B and D) in yeast (plots A and B) and human (plots C and D) PG _0.001&0.0014_ networks. The gold standard dataset used, KG≥2 evidences, is described in the section: Validation of the second order predictions for the PG networks.

Comparison of the association profiles identified 1,668,584 protein pairs in yeast and 49,117,115 protein pairs in human sharing at least one third of their interacting proteins in the PG_0.01&0.014_ network matrices. The similarity scores of the profiles were validated using the different KG datasets i.e. Int, Kegg, Goss, Foss, Reactome, and Reactome_int (see Figure S14, Figure S15, Figure S16, Figure S17, Figure S18, Figure S19, and Figure S20 in Text S1) and the integrated and refined KG≥2 evidences dataset (Figure 4). Bits and specific bits similarity scores (see the section: Second order predictions from the PG networks: Measuring the similarity of protein interaction profiles) positively correlate with an increase in precision for all the KG datasets (see Figure S14, Figure S15, Figure S16, Figure S17, Figure S18, Figure S19, and Figure S20 in ) and the refined KG≥2 dataset (Figure 4).

Bits and specific bits scores show very similar behaviour in all the KG datasets most probably due to the large set of potential random interactions in both PG matrices that make it very unlikely that two proteins would share a significant number of interactions by chance (see section 18 in Text S1).

First order predictions based on Fisher scores yielded about 90,000 predictions in yeast with a precision≥80% (see Figure 1), while second order predictions only yielded 95 predictions at the 80% precision level in the KG≥2 validation dataset (Figure 4b) and 8,390 predictions maximum in the single evidence KG datasets (Kegg validation recall in yeast; see Figure S18b in Text S1). The same observation is valid for human with about 1,000,000 hits at 80% precision level in the Fisher first order predictions and only 889 second order predictions at 80% precision in the KG≥2 validation (Figure 4c) and a maximum of 118,800 predictions in the single KG datasets (Reactome validation recall in Figure S19f in Text S1). Since second order predictions are predictions performed over first order predictions, there is likely to be an accumulation of second order error over the primary error, lowering the general performance. Nevertheless, a common observation in all the validations is that the PG_0.001&0.0014_ networks have second order functional information of real biological value absent in the first order predictions. Although, using context does not predict many more interactions, this analysis is important because it confirms that the topology of our predicted network has real biological meaning.

## Discussion

The scoring functions of the three *ab-initio* methods (GECO, CODA and hiPPI), showed close correlation with precision in predicting true, functionally associated proteins (Figure 1). The correspondence of the p-value scoring functions with prediction reliability and also the complementary nature of the prediction datasets, suggested by their independent mutual information, enabled the Fisher integration method to perform well. Fisher meta-statistic, untrained, integration of the four datasets (GECO, CODAcath, CODApfam and hiPPI) yielded a significant increase in prediction power within the yeast and human proteomes, adding value beyond any single method or the sum of all of them. Fisher integration thus allowed us to build comprehensive PG_0.01&0.014_ integrated models independent from the KG data, and at highly reliable precision levels (80%) for yeast and human.

While the KG network models contain much of the current knowledge on protein functional associations provided by disparate experimental resources, in yeast and human, the PG models represent sets of predictions inferred by the integration of different *ab-initio* (non-experimental) methods. Experimental (KG) and predicted (PG) networks share all of the main topological features explored in this work. In summary the node k_i_ degree distribution, assortativity, clustering distribution, and clustering average coefficient for each of the PG and KG networks demonstrate a trend towards a scale-free organization as network confidence increases. KG and PG are both non-random networks, both in the connectivity and in the statistical weight distributions of their edges (Figures 2 and 3; sections 8–13 in Text S1).

Different data integration methods are applied for reducing noise (error) in the KG and PG models, thereby generating analogous frameworks for the KG and PG models built at different reliability levels. In the KG models the associated error is inversely correlated to the number of evidences supporting a given protein-protein association. Reducing error by summing evidences is analogous to the repetition of experiments carried out in standard experimental protocols [3]. In the PG models, the Fisher method reduces noise by integrating the weighted (p-value) evidences within a probability space which has finer resolution than the presence/absence binary space used in the KG models. Gaussian distributions, typical of random network topologies, appear in the high node (k_i_) connectivity part of the plots for the least reliable KG and PG models, disappearing in the KG and PG models built at higher levels of reliability (see Figures 2 and 3). This indicates that errors in determining true protein associations are common to both KG and PG models and that the KG and PG network topologies respond in the same way to analogous methods for reducing noise (data integration). We also observed that the topology of the PG_0.01&0.014_ models have functional information of real biological networks beyond first order binary predictions (see Figure 4; and section 18 in Text S1).

Since one of the prediction methods, hiPPI, exploits available experimental data by inheriting experimentally validated interactions between homologous proteins there may be some concern that the dependency of the hiPPI predictions on some of the KG datasets could bias the PG network models so that the features resemble those of experimental KG networks. Addressing this possibility we repeated the main analyses of this work excluding the hiPPI predictions and demonstrated that the similarity of the PG and KG models remained and is therefore not due to any circular information or bias. This confirms our previous observations and conclusions of our work (see section 21 in Text S1).

Coverage of reliable PG_0.01&0.014_ predictions by KG datasets appears much higher in yeast (18%) than in human (1.34%) for all the analysed cases (Table 1), highlighting the better network characterisation in the yeast proteome network. 82% of the predicted associations in yeast were not backed by any KG data indicating considerable dark matter in the yeast PG network. These figures are even higher for human; where a 98.5% of predictions are absent from the KG databases. Although low overlaps between high-throughput experimental datasets is not a surprising observation, the relative differences in the amount of dark matter found for yeast and human hint at important differences in the progress of our knowledge of these two organisms' PPI networks. The dark matter in the PG models of yeast and human contains hubs (i.e. dark hubs) which are key for network integration and functioning and which seem to be involved in disparate functions in both organisms (Table 2). In yeast dark hubs include many membrane embedded proteins with unknown functions. Membrane proteins are usually more poorly characterised than soluble proteins, due to the current design of experimental techniques, and therefore prediction methods could assist in characterising associations for these proteins.

For human, the top ranked dark hub dataset (see *n* column in Table 3) is significantly enriched in kinases and GTPase/Ras regulatory proteins associated with important biological regulatory pathways. These results reveal the existence of key regions (i.e dark functional regions) belonging to protein network functional space that are poorly characterised by experimental sciences but highly represented in the PG models. As for the membrane proteins in yeast, predictions of these proteins would be helpful in identifying associations which currently elude experimental approaches. It is quite well known that current experimental high-throughput datasets show limitations with respect to coverage and also systematic errors. For example, Y2H does not perform well on membrane-associated proteins and transient interactions tend to be under-reported [34]. This observation agrees with our analyses, which shows that dark hubs are particularly enriched in integral membrane proteins and transient interactions such as those involved in kinase mediated regulation, a mechanism over-represented in the Ras signalling pathway.

Dark matter may even be more extensive than suggested by the initial comparison of PG and KG models. KG and PG models both show a non-hierarchical structure, as shown by the clustering parameter distribution (Figure S11 in Text S1), whilst preserving a highly modular structure (Table S3 and Table S4 in Text S1). Since all the functional modules must ultimately be integrated within a functioning organism, the high modularity and non-hierarchical structure suggests that our PG and KG models are incomplete lacking proteins (nodes), and protein-protein associations (edges) still uncharacterised in our KG and PG models.

Since much of the PG network is dark matter containing hubs and other important functional regions not easily reached by current experimental designs (especially in more complex organisms like human), and since the PG models show the most important properties of real, biological networks, resembling the properties observed in the KG models, we can conclude that the yeast and the human PG networks are valuable models, akin to the currently more accepted KG models, for investigating the properties of real biological networks, complementing and completing experimental studies in Systems Biology.

## Materials and Methods

### *Ab initio* methods used for building the Predictograms (PG methods)

**Overview of the Methods.** Homology inherited Protein-Protein Interaction (hiPPI) method, scores potential protein-protein interactions based on their homology to known interacting protein pairs; Co-Occurrence Domain Analysis (CODA) method, looks for and scores protein pairs in a given target genome (e.g. yeast or human) found as fused (Co-Occurring) domain architectures in homologues from genomes of 575 different species; Gene Expression COmparison (GECO) method, measures the correlation of gene expression profiles between protein pairs (detailed explanation of the *ab initio* methods in section 1 in Text S1).

### Running the PG methods on the human and yeast proteomes

The GECO, hiPPI, CODAcath and CODApfam methods were run against all sequences in the human (*Homo sapiens*) and yeast (*Sacharomyces cerevisiae*) proteomes (detailed datasets information in section 19 in Text S1). Proteome files were downloaded from the Integr8 database June 2007 (section 19 in Text S1). GECO retrieved 26,292,126 protein pairs of predictions for human and 10,371,735 for yeast with total sequence coverage of 21% and 81.5% respectively. hiPPI yielded 86,099 protein pairs of predictions for human and 12,070 for yeast, with total protein sequence coverage of 31% and 56.6% respectively. CODAcath yielded 32,259,881 and 678,928 predictions (coverage 39% and 36.4%) for human and yeast respectively. Whilst CODApfam generated 24,984,943 and 336,781 predictions (coverage 57% and 58.4%), for human and yeast respectively.

### Calculating p-values for the predictions and data integration

#### P-value calculation

A score for the cumulative frequency distributions was calculated for each of the four prediction datasets (GECO, hiPPI, CODAcath and CODApfam) using the curvefit tool from MATLAB. The particular Probability Density Functions (PDF) associated with the score distributions for each of the four methods was calculated in order to translate the scores into p-values. Right tailed Ztests were performed to ensure that the PDF distributions of the PG datasets fit random Gaussian distributions with different means μ (null hypothesis) at 5% significance level for accepting the null hypothesis being false (see section 20 in Text S1). Generally, the p-values constitute a normalised measurement for comparing the performance of different PG methods.

#### Statistical dependence between the prediction datasets

Mutual information was calculated between the prediction datasets, to detect potential dependencies. The small values calculated for the mutual information (or conditional dependency) between pairs of predictors, indicated that the datasets were largely conditionally independent (Table S1 in Text S1).

#### Integrating the prediction data

The p-values from each method were integrated using two methods: Simple integration, and Fisher weighted (Fisher_W) [12]. The simple integration method was done by selecting the most significant prediction (lowest p-value) from all the prediction methods. Fisher_W formula introduces a weight correction -*w_i_*- to sum prediction p-values -Ln(p_i_)- in order to maximize the overall statistical power.
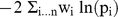



The weights for the Fisher_W method were calculated using a MATLAB script. This consisted of simultaneously running a *Monte Carlo Method of 5^th^ order* [46], [47] and *Enhanced Simulated Annealing (ESA)* [48] functions. The weights were calculated so as to maximize the statistical power and confidence. Normalised p-values were calculated based on the score distributions of the integrated methods (Simple_and Fisher_W datasets) using the same methodology explained above for the independent PG datasets. The whole PG matrix in yeast contained 10,642,398 pairs and in human 70,908,243 pairs.

### The GO Semantic Similarity refined dataset (Gossr) used for validating the prediction methods

We benchmarked our predictions using the highest quality annotations of yeast and human proteomes in the Gene Ontology (GO) database [26]. GO provides annotation codes which enable the selection of protein annotations based on quality and evidence source (see further details in section 14 in Text S1).

The GO terms' Semantic Similarity (Goss) scores were calculated for all versus all protein pairs in human and yeast proteomes as described by Lord et al. 2003 [49], using the GO relational graph implicit in the GO ontology file (GO ontology files; OBO v1.0 format 30th-October-2008; http://www.geneontology.org/). Sets of protein pairs with significant Goss score (Goss≥4.0; [50]) in the refined sets of GO annotations were selected as validating datasets for the yeast and human protein pair predictions. These protein pair sets are referred to Goss refined (Gossr) datasets.

### Precision and recall calculation

Precision was calculated as the ratio of accumulative TP/TP+FP at different prediction p-values, where TP (True Positives) is the rate of hits predicted within the validation dataset of true protein binary associations (e.g. Gossr, see section above), and FP (False positive) is the average rate of hits predicted from 1000 random models of the same validation dataset.

The FP are the randomly selected PPIs above different scoring thresholds (i.e. prediction p-values). The FPs are calculated as an average of 1000 random validation iterations to estimate the errors (deviations) associated with the calculation. We then compare the relative differences in the TP and FP rates in the ranked prediction list, obtained by using our predictor and a random approach. For example, a precision ≥90% associated with a p-value≤0,001 means we find 9 times more TPs in the set of predictions with p-value≤0,001 than a random predictor does by chance. In our analyses the precision (ie TP/TP+FP) will always tend to 50% because we select the same number of FPs from our random predictors as given by the integrated prediction method.

Using a random model for benchmarking it is possible that a randomly selected PPI could be a known TP, by chance, although the probability is expected to be very low since the space of known PPI (TPs) is much lower than the space of random PPIs pairs considering all possible combinations. It is also likely that any of the gold standard datasets, or combinations of them, do not contain all the true PPIs taking place in nature. Therefore it is not possible to correctly estimate FPs in the ranked predictions, based on pairs absent in the validating datasets (ie many of these FPs may be currently uncharacterised TPs). In any case, the consequence of considering TPs as FPs in the random validation model used in this work is conservative, giving an underestimate of the performance of our predictor (see section 2 in Text S1).

Although recall is usually defined as the TP/(TP+FN) ratio, since not all the true PPI are known in our validation model, we can not reliably estimate the FN rates. Therefore, in this work Recall is calculated as the accumulated number of predicted hits by a given method, at different p-value levels.

### Predictogram (PG) construction

Yeast and human PG protein networks were built based on the binary protein prediction data selected at different discrete Fisher_W p-value statistical significance levels. Fisher_W predictions were chosen because these gave the best results from the benchmarking. Various PG networks were generated over a range of predicted p-value cut-offs. The p-value cut-off used to generate a given PG network is specified in the subscript of its name. For example if a p-value cut-off ≤0.01 was used the PG network was termed PG_0.01_.

### Knowledgegram (KG) construction

The construction of KG protein networks for human and yeast proteomes was based on the existence of protein functional links. For the interaction databases HRPD [30], MINT [29], Intact [28] evidence of a protein interaction gave an evidence score of 1. For the pathway resources, Reactome [24] and Kegg [25], shared pathway membership was sufficient for an evidence score of 1. An extra Reactome_Int dataset was built based on physical protein interaction evidence in Reactome. Binary protein associations in GO and FunCat were identified using the Semantic Similarity score calculated using the ontology association graphs in GO and FunCat [27] respectively. For sets based on GO (Goss datasets) we used all the annotations in GO in order to maximise coverage of GO functional space within the KG networks. These Goss datasets are therefore expected to contain more noise that the refined Gossr datasets used to validate the methods (see PG methods' validation section above). Semantic similarity values were calculated with the Resnik method [49], [51] as described in the section above: The GO Semantic Similarity refined dataset (Gossr) used for validating the prediction methods. Sets of protein pairs with significant functional associations (Goss≥4.0; [50]) gave a score of 1. A Foss (FunCat semantic similarity) significant set was obtained from the FunCat [27] database. Foss score was calculated using the same process as GO with the Resnik method. Int dataset was generated by the union of all the above datasets excluding Goss, Foss, Kegg and Reactome.

A cumulative score was associated with each edge (functional link) to represent the number of independent resources with evidence of the functional link between the two proteins. The KG models statistics are shown in Table S5 in Text S1.

### Network randomisation

Two different randomisation procedures were implemented. The first method randomised the p-values associated with edges in the PG network and the # of evidences associated with edges in the KG models, whilst keeping the same pairs of connected nodes in the matrices. These models are referred to as *p-values random models* and they were built to analyse the distribution of the statistical weights associated with protein edges (p-values and # of evidences related to edges) compared to random behaviour. The second randomised model, referred to as the *adjacency random model*, was generated by randomly distributing all nodes, p-values and # of evidences in the PG and KG pairs-wise datasets. Any new self-associations in the PG network datasets produced by the randomisations were removed. The *adjacency random models* were built to analyse the distribution of edges and p-values in the KG and PG models compared to random behaviour. Both models went through 1000 randomisation iterations.

### Network topology structure characterisation

In order to compare the PG/KG networks generated by this study several different network statistical features were calculated. Topological parameters included the node degree connection (k_i_) [37], [38], degree correlation (assortativity) [37]–[40], clustering distribution [41], [52] and average clustering coefficient [37]. Distance based metrics to characterise the networks included the characteristic path length ℓ [37], radius, diameter and eccentricity [42] (see section 7 in Text S1).

### Calculating the PGk_i_ enrichment ratio and the PG functional enrichment

In order to determine whether some nodes had elevated degree connections in the PG, the relative enrichment of the node degree connection (k_i_) for nodes in the PG network compared to the KG network was calculated for all the nodes (proteins) using the following formula: ***PGk_i__er (p_i_) = (PGk_i_**−**KGk_i_)/(KGk_i_**+**1)*** where ***PGk_i__er*** is the PGk_i_ enrichment ratio of the protein ***p_i_***, ***PGk_i_*** *is* the k_i_ value of the protein **p_i_** in the KG network and ***KGk_i_*** is the k_i_ value of the protein **p_i_** in the KG network. Yeast and human proteins were ranked using the PGk_i__er parameter values and the ranked lists were used as input for the GOrilla web server (http://cbl-gorilla.cs.technion.ac.il/). GOrilla is a tool for identifying and visualizing enriched GO terms in ranked lists of genes (Eden et al. 2009, [45]).

### Second order predictions from the PG networks: Measuring the similarity of protein interaction profiles

For each protein pair, the vectors of interacting proteins, within the PG_0.01_ in yeast and the PG_0.014_ in human network matrices (0,01 and 0,014 cut-offs relate to 80% precision in yeast and human respectively), were compared using different similarity measures, such as: bits, specific bits and congruence. These similarity scores, *which are calculated over the PG network matrices*, are termed *second order predictions* (see section 17 in Text S1).

The bits score formula is b(p_1_,p_2_) = b_1_, where p_1_ and p_2_ are the two proteins compared and b_1_ is the number of shared interacting proteins between the two proteins' interaction vectors in a given PG network matrix. The specific bits score was calculated using the following formula: s(p_1_,p_2_) = b_1_·[−log(b_1_/(b_1_+b_2_))], where p_1_ and p_2_ are the two proteins compared, b_1_ is the number of shared interacting proteins, and b_2_ is the number of non-shared interacting proteins between the two compared proteins in the PG networks. Congruence is a similarity measure between pairs of protein interacting vectors that was calculated as described in Lehner [53]. Bits and specific bits scores were calculated for the yeast and human PG networks; whilst congruence calculation was only performed for yeast since the size of the human PG_0.014_ network matrix (13,961×13,961, see Table S5 in Text S1) was too large to make it feasible to implement the combinatorial calculation implicit in the congruence measure.

### Validation of the second order predictions for the PG networks

Second order predictions were ranked based on the different similarity score values (see section above) from the most significant to the least significant. Validation was performed using as true positives (TP) protein pairs from the KG matrices in yeast and human respectively (Int, Goss, Foss and Kegg in Yeast and Goss, Foss, Kegg, Int, Reactom_Int, and Reactome in Human; see the section above: Knowledgegram (KG) construction) mapped to pairs in the ranked lists. An extra gold standard dataset of mapped true positive hits was built using those pairs present in two or more KG datasets (KG≥2). False positive (FP) sets were obtained by mapping the same KG gold standard datasets on randomised lists of second order predictions ranked lists, with 1,000 random iterations in yeast and 500 in human (fewer times in human balancing the sample size against computational cost).

Precision and recall parameters were calculated as described above, the precision mean and error (standard deviation) values were calculated based on the TP and the different accumulated random FP distributions. In order to present representative results values with standard deviations more than 1/3 of the mean were ignored, as they were due to the small size of the TP and FP samples at the beginning of the accumulated distributions (for further details see section 18 in Text S1).

## Supporting Information

Text S1 Supporting information.
(4.01 MB PDF)
