# Supplementary material for: Finding the “Dark Matter” in Human and Yeast Protein Network Prediction and Modelling
Source: PLoS Comput Biol. 2010 Sep 23;6(9):e1000945. doi: 10.1371/journal.pcbi.1000945 (PMC2944794; doi:10.1371/journal.pcbi.1000945)
Supplement: Text S1 — Supporting information. (4.01 MB PDF) [file pcbi.1000945.s001.pdf]

Finding the “Dark Matter” in Human and Yeast Protein Network Prediction and Modeling

Supporting Information

1. *Ab initio* methods used for building the Predictograms (PG methods).

GECO (Gene Expression COmparison) Method: Microarrays provide a high throughput approach for identifying functionally related proteins. For the current evaluation in yeast we chose a compendium of publicly available gene expression data from the Eisen lab. The dataset consisted of 80 experiments broadly corresponding to a previously published dataset [54]. A clear signal between microarray data and interacting proteins has previously been observed with the yeast dataset and the MIPS (MPACT) PPI dataset [55]. For human we use the E-TABM-185 compendium dataset of 6000 gcrma normalised HGU133-A affymetrix microarrays assembled by array-express [56]. A maximum of 5 values were allowed to be missing from a given genes expression profile, using the C-clustering libraries masking function. Because virtually all yeast ORFS are present on the microarray, the combination of all pairwise correlation coefficients represents a near total population of ORF pairs. For the human hgu133a affymetrix chips 14,500 genes are well characterised giving a very large set of similarity scores.

CODA (Co-Occurrence of Domain Analysis) Method: CODA is based on domain fusion analysis. The aim of gene fusion methods is to infer protein-protein interactions or more generally functional associations between pairs of separate protein chains in a genome of interest whose orthologues have become fused in another species. Enright et al. (1999) and Marcotte et al. (1999) [57,58] were the first groups to introduce this approach. CODA uses a Multi-Domain Architecture (MDA) representation of proteins in complete genomes (target genomes) provided by Gene3D Multi-Domain Architecture datasets [59]. The Gene3D database contains protein sequences for all complete genomes with predictions for CATH [22] and Pfam [23] domains as well as functional annotations including GO. MDA CATH and PFAM datasets were created from 527 complete genomes (50 eukaryotes, 438 eubacteria and 39 archaea), CODA predictions were performed on these two (CATH and PFAM) datasets.

CODA scoring method: Here we consider how the method is implemented for a particular pair of proteins $i = (p,q)$ in a query genome g. P is the set of domains in protein p. $a \in P$ denotes that protein p contains a domain of superfamily a. J is the set of domain pairs $j = (a,b)$ where $a \in P$, $b \in Q$. In other words J consists of all the distinct pairs of domains between proteins p and q. It is also required that $P \cap Q = \{\}$, as the two proteins must not share any domains of the same superfamily.

To determine a fusion event we require that a target genome (one other than the query genome) contains a protein s where $a \in S$ and $b \in S$ i.e. domains which are separated in the query genome are found fused in the target genome. The set T comprises those

genomes other than *g* which contain such proteins *s*. For a domain pair *j* in genome *g*, the fusion score C_j is taken as a maximum over all genomes in *T*:

$$C_j = \max_{t=1}^{|T|}\left(\frac{1}{n_{g_A}+n_{t_A}}+\frac{1}{n_{g_B}+n_{t_B}}\right) \quad (1)$$

Where |*T*| is the number of elements of set *T* (i.e. the number of target genomes), n_{g_A} and n_{g_B} are the frequencies of domain *A* and domain *B* respectively in genome *g* and n_{t_A} and n_{t_B} are the frequencies of domains *A* and *B* respectively in genome *t*. For a particular protein pair *i*, in query genome *g*, the maximum C_j is taken over all possible domain pairs *j*.

$$C_i = \max_{j=1}^{|J|}(C_j) \quad (2)$$

Where |*J*| is the number of elements in set *J* (i.e. distinct domain pairs). Thus C_i is the CODA score for proteins *p*,*q* (pair *i*); the best (highest) score over all domain pairs between the proteins and over potential fusion proteins in all genomes (other than the query genome). The important novel aspect of this score is that it takes the maximum score over all the genomes whereas other methods do not consider target genomes individually. The score was chosen to reflect the uncertainty that fused domains and their unfused relatives are orthologues. The highest (best) possible score is 1 which is returned when there is only one example of each domain family in the query genome and one fused protein in a target genome, with no other domain homologues. In this case it is highly likely that the query protein domains are orthologous to the target protein.

hiPPI (homology inherited Protein-Protein Interaction) Method: The hiPPI method takes advantage of the Gene3D families of structurally conserved proteins as well as multiple sources of protein-protein physical interaction (PPI) data to reliably infer ('inherit') novel protein-protein interactions from homologues. hiPPI exploits the Gene3D protein families (G3D_families) datasets [59]. These are families of proteins with similar multi-domain architectures generated using an automated, but conservative, clustering procedure. Interactions are only inherited between proteins belonging to the same Gene3D family, even though there may be recognisable sequence similarity with a protein in another cluster. This step helps to reduce the amount of noise produced by attempting to inherit from overly-distantly related sequences.

The interaction dataset is formed from a merger of the PPI resources from MIPS, IntAct, HPRD and MINT protein-protein interaction datasets, obtained from the Gene3D database [59]. From each dataset the interacting proteins, their family, species, and experimental method was retrieved. Gene3D family codes consist of 11 elements. The first is the root family code, then the family is further sub clustered at 10 levels of sequence identity, termed S-levels (S30, S35, S40, S50, S60, S70, S80, S90, S95, S100).

hiPPI score (for a graphical description see Figure 1): For every test protein ('the inheritees'), all the relatives ('the inheritors') with interactions with proteins ('the complementors') with relatives in the inheritee's species ('the complementees') are identified. The inherited interaction ('inheritance') is that between the inheritee and the potential complementees. Known direct interactions were discarded. For each inheritance the similarity between the inheritee and the inheritor is measured by what Slevel they belong to, on a scale of 1 – 11 (1 is the family code and 11 is 100% identical); this is termed the 'iLevel'. Identically, the 'cLevel' is calculated for the complement. The two values are then averaged to create the 'icLevel'.

At this stage two alternative steps can be taken, and both are useful in different situations. The first assumes that if a protein interacts with one member of family then it is likely to at least show some affinity for another member in the same species. This can be considered biologically realistic, as the effect is seen in many genetic experiments (i.e. complementation tests). In this case all inheritances are counted. The second disregards this assumption in order to identify the probable biologically most important interaction. In this case, those inheritances with either a cLevel or an iLevel of 10 are disregarded. For the current study the former approach was used.

Since each protein-protein interaction can be inherited from more than one species, experimental method or iLevel, a summed score is created (the 'iScore') for each distinct pair of icLevels (NB iLevel = 10, cLevel = 8 is not the same as iLevel = 8, cLevel = 10). The first entry (non-redundant experiment) at that level contributes the full score of the icLevel. For subsequent entries at that icLevel if the experiment type or species is not new the score is halved; if neither is new but is a recombination of previously observed ones then the score is quartered. The final iScore is the sum of all the intermediate icLevel scores.

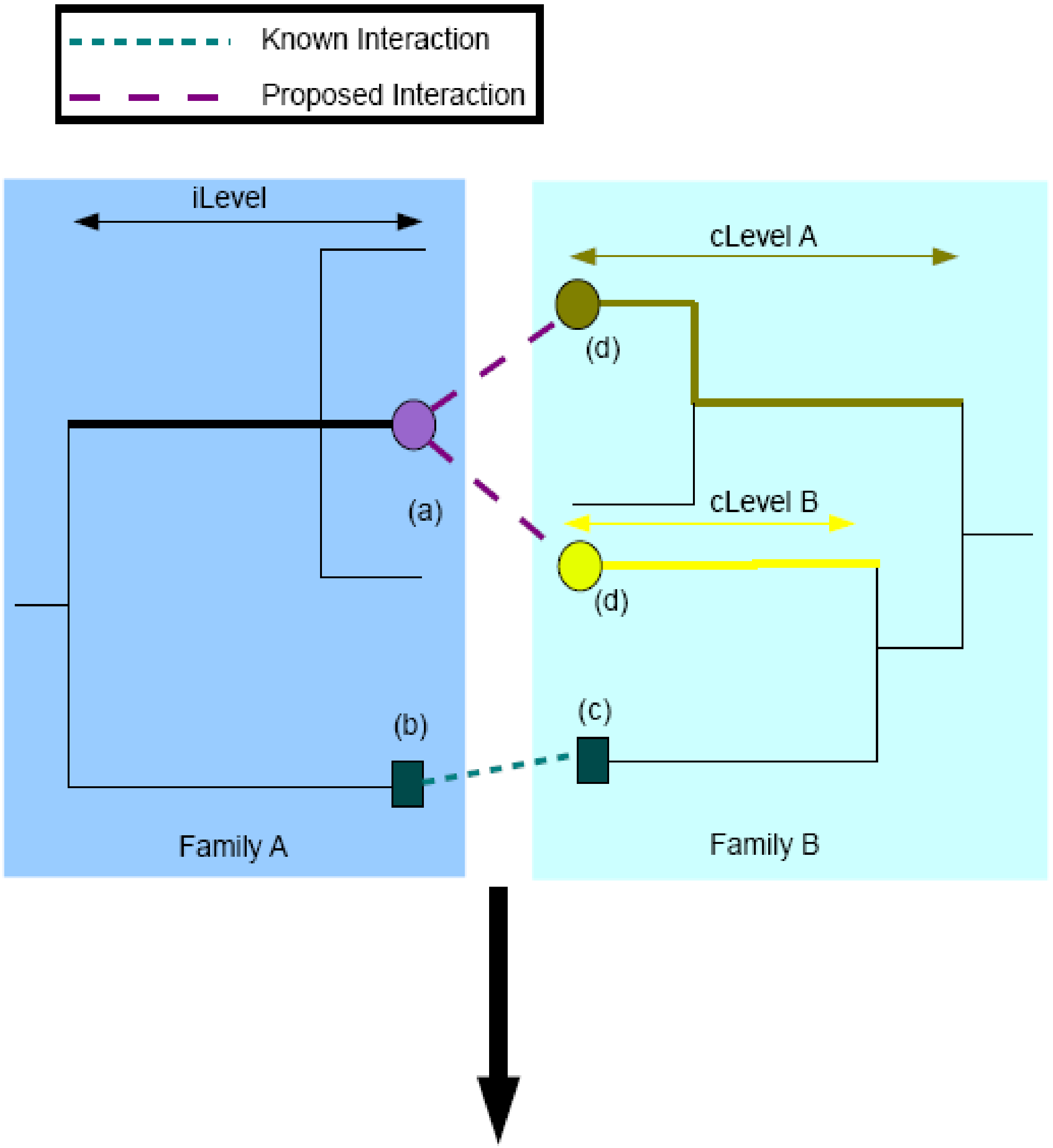

Interactors	iLevel	cLevel	icLevel	Species	Experiment	Adjusted Score	iScore
ProtA-ProtB	8	9	8.5	4932	2-hybrid	8.5	
ProtA-ProtB	8	9	8.5	4932	Anti-tag coip	4.25	
ProtA-ProtB	4	6	5	40674	2-hybrid	5	
ProtA-ProtB	1	1	1	4932	Tap	1	
ProtA-ProtB	1	1	1	10116	Tap	0.5	19.25
ProtA-ProtC	8	4	6	4932	Pull down	6	
ProtA-ProtC	8	4	6	4932	2-hybrid	3	
ProtA-ProtC	4	1	2	7227	2-hybrid	1	10

Figure 1. The hiPPI approach. (A) An example of identifying two potential interaction partners (labelled (d)) for the query protein (a). The interaction is inferred from the known interaction between (b) and (c), which are homologous to (a) and (d) respectively. Small example trees are shown for each family; each branch in the trees occurs at a particular family S-level (i.e. 80% sequence identity). The S-level in common between (a) and (b) is the iLevel, while the S-levels in common with (c) and the (d) s are the cLevels.

2. Benchmarking studies for the integrated prediction methods using KG datasets.

These studies explored the variation of Precision vs. Recall with progressive reduction of noise, due to increasing evidence (yeast ≥ 2, 1 evidences and in Human ≥ 3, 2 and 1), in the gold standard KG datasets used to estimate statistical power. These plots (Figures 2A and B) reinforce the results showed in Figure 1 in the main manuscript by showing that reduction of noise in the gold standard KG datasets increases the area under the curve in the precision vs. recall plots for both species.

There are two related effects that could distort the estimation of FP rates in our validation model: the fact that the gold standard datasets do not contain all the true PPIs in nature; and the fact that the random model used for benchmarking can randomly select true positives. Therefore, we have performed an experiment to estimate the consequence of considering TPs as FPs in our validation protocol.

We have validated performance using as random datasets the combination of 2/3 randomly obtained PPI plus a random selection of 1/3 known TPs from the GOSS, Int and Reactome_int datasets (these TP enriched random datasets are called “noisier”). We then compare the results for these noisier datasets with validations performed using the remaining 2/3 of PPIs from the same gold standards (Figure 2C and D). When TPs are counted as FPs the precision decreases in all cases giving an underestimate of the performance of the prediction methods.

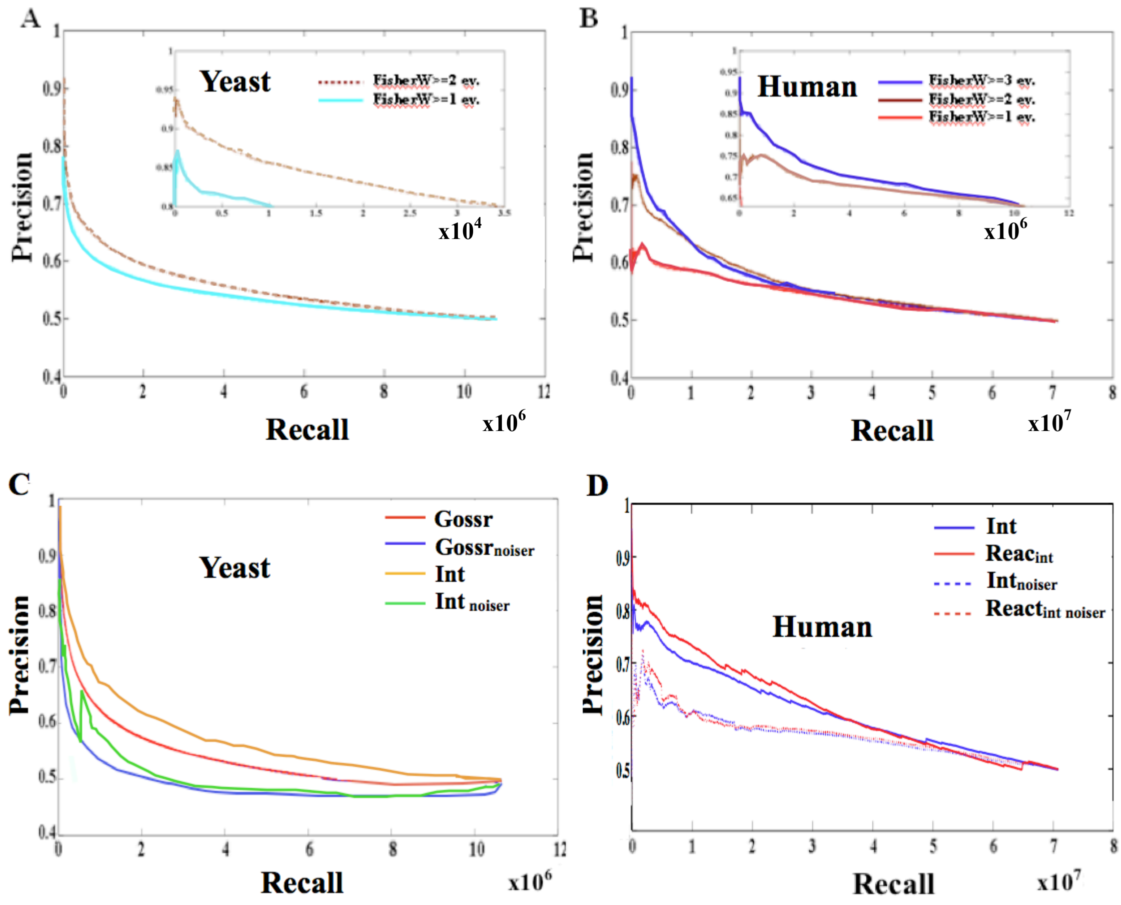

Figure 2. Prediction power of the integrated methods in yeast and human. Panels present precision versus recall for Yeast and human, as discussed in the main manuscript. In panel A, validation for yeast was performed using KG ≥2 evidences and KG ≥1 evidences as gold standards. Panel B shows the validation in human using the KG ≥3, 2 and 1 evidence gold standard datasets. The enlargement highlights the improvements obtained. In panel C validation was performed in yeast using the Goss and Int noisier datasets as random models and the remaining 2/3 of the respective gold standard datasets as TPs. In panel D validation was performed as in panel C but in human using the Reactome_int extra datasets.

3. Metrics and Mutual Information

A. Metric for Yeast

**Yeast**	**GECO**	**CODAcath**	**CODApfam**	**hiPPI**
**GECO**	-	0.9982	0.9987	0.9697
**CODAcath**			0.9697	~1
**CODApfam**				~1
**hiPPI**				

B. Mutual information predictions in Yeast

**Yeast**	**GECO**	**CODAcath**	**CODApfam**	**hiPPI**
**GECO**	-	0.0018	0.0013	5.4612.e-05
**CODAcath**			0.0059	9.3768.e-07
**CODApfam**				9.2720.e-07
**hiPPI**				

C. Metric for Human

**Human**	**GECO**	**hiPPI**	**CODAcath**	**CODApfam**
**GECO**	-	0.9998	0.9326	0.9311
**hiPPI**			0.9999	0.9998
**CODAcath**				0.9987
**CODApfam**				

D. Mutual information predictions in Human

**Human**	**GECO**	**hiPPI**	**CODAcath**	**CODApfam**
**GECO**	-	1.1143.e-04	0.0560	0.0585
**hiPPI**			7.5174.e-05	1.2897.e-04
**CODAcath**				0.0011
**CODApfam**				

Table 1. Study of dependencies between the prediction methods. The mutual information remains low suggesting a minimal overlap of features. Tables A and C correspond to the normalised measure or universal metric whilst B and D show the mutual information scores between the predictors. In order to meet the statistical requirements for integrating PG datasets it is important to ascertain whether

integration improvements could be an artefact caused by correlation and dependencies between the prediction methods. Mutual information is a more general measure of correlation than the Pearson's correlation coefficient metric, capturing both linear and non-linear correlations.

Metric

Many applications require a metric, that is, a distance measure between points. The quantity $d(X,Y) = H(X,Y) - I(X;Y)$ satisfies the basic properties of a metric; most importantly, the triangle inequality, but also non-negativity and symmetry. Where H is the entropy between X and Y samples and I the corresponding mutual information.

In addition, one also has

$$d(X,Y) \leq H(X,Y),$$

and so obtains

$$D(X,Y) = d(X,Y) / H(X,Y) \leq 1$$

The metric D is a universal metric, in that if any other distance measure places X and Y close, then D will also judge them close.

In this way, mutual information and mutual metric measure opposite behaviours. The latter is adopted with the aim of normalizing for database size.

4. Validation of the FisherW p-values using physical interaction datasets.

We have performed the validation of the Fisher predictions using the “Int” datasets for yeast and human and with an additional Reactome_int dataset for human (see Figure 3). The Int dataset combines the interaction data from the HRPD, MINT, and Intact databases (see Methods). In human we have used an extra Reactome_int dataset which contains the physical interactions annotated in the Reactome database. No similar Reactome_int dataset was available for yeast since Reactome is only focused on human molecular interactions and reactions. The methodology employed for performing analysis of precision vs. coverage was the same as that described in Methods.

We found that Fisher p-values correlate inversely with the precision scores in the yeast and human validations (see Figure 3a and b respectively), as expected if physical interaction information is linked to the Fisher prediction score. Fisher integration retrieves 240,840 predictions with precision ≥ 80% from the Int validation dataset in yeast (Figure 3a). This figure is more than two fold the number of hits obtained in the Gossr validation at the same precision level ≥ 80% (see Figure 1 in the main manuscript). For the functional analysis performed in our work, physical interaction validation with the Int dataset in yeast assigns a precision ≥ 90% to the $PG_{0.01}$ dataset (pairs with p-values ≤ 0.01), higher than the Gossr validation estimation which assigns a lower precision (precision ≥ 80%) to the same $PG_{0.01}$ dataset (see Figure 1a in the main manuscript).

For the human Int validation dataset Fisher predicts 455,410 pairs with precision ≥ 80%,

whilst in the validation using Reactome_int the number of predictions rise to 3,823,840 at the same level of precision ≥ 80% (Figure 3b). These figures are about half and almost four fold, respectively, the $PG_{0.014}$ human dataset used for functional analysis in our work. In the Gossr validation of the $PG_{0.014,}$ the selected Fisher predictions achieve precisions ≥ 80%. However, in the Int and Reactome_int validations the $PG_{0.014}$ selected Fisher predictions dataset reaches precisions of ≥ 76% and ≥ 82%, very close to the ≥ 80% precision assigned by the Gossr validation of the same $PG_{0.014}$ dataset.

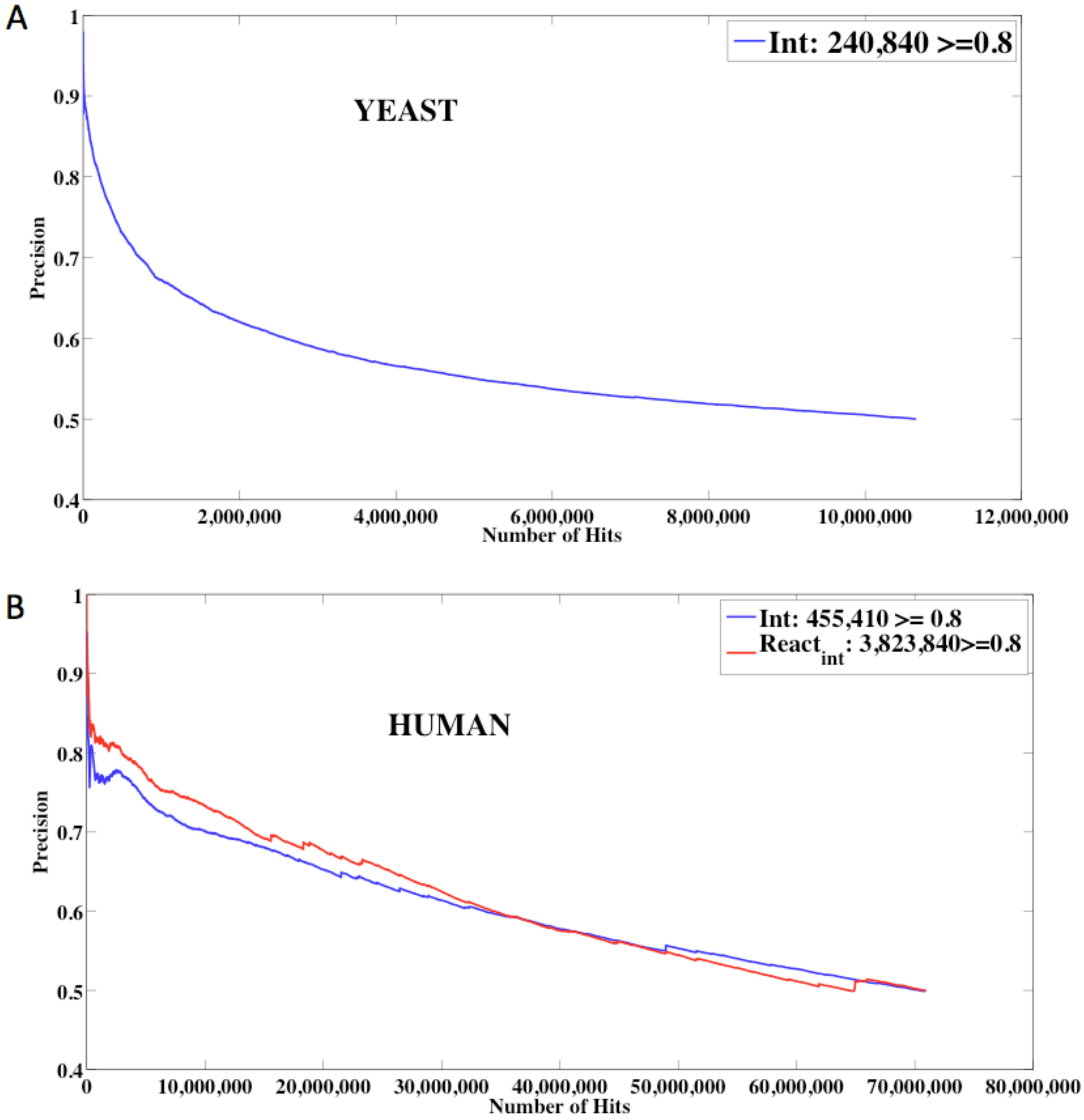

Figure 3. Prediction powers of the Fisher integrated method in yeast and human using datasets of pairs of physically interacting proteins. Panels present the results for yeast and human in terms of precision (y-axis) versus recall (x-axis), as discussed in the main manuscript. Panel A shows the validation in yeast using the Int dataset (a combination of the physical interaction datasets; see Methods) as gold standards (blue line). Panel B shows the validation in human using the Int (blue line) and Reactome_int (red line) gold standard datasets. The boxes highlight the number of predictions retrieved with precision ≥ 80%.

5. Fisher integration of the STRING individual *ab-initio* prediction datasets and benchmarking of the integrated STRING predictions.

We performed a weighted Fisher integration of the neighbourhood, fusion, co-occurrence and co-expression prediction datasets obtained from the STRING site (http://string-db.org/newstring_cgi/) [14]. The yeast and human STRING predictions were extracted from the downloaded file: protein.links.detailed.v8.2.txt.gz. Duplicated (ie redundant) protein-protein pairwise predictions were identified in the STRING yeast and human datasets and removed. The four STRING prediction datasets scores showed Gaussian distributions and their scores were translated into p-values. Then, FisherW integration was performed for the STRING prediction datasets following the same protocol implemented in this work for obtaining the PG predictions (see "*Calculating p-values for the predictions and data integration*" section in Methods).

We compared our PG Fisher prediction performance against the STRING Fisher integrated scored predictions. STRING is an updated and well-known resource for predicting protein interaction, and represents a good gold standard to compare against. STRING comprises, amongst other methods, four *ab-initio* prediction methods similar to the methods integrated in the PG models. Amongst other methods, STRING provides the following *ab-initio* predictions based on genome and gene expression comparison: gene neighborhood, gene fusion (comparable to CODA), gene co-occurrence, and gene co-expression (comparable to GECO). STRING does not include a prediction algorithm similar to hiPPI, but instead STRING implements a phylogenetic profiling-like method (gene co-occurence) and a gene genome co-localization method (gene neighborhood). In any case, hiPPI predictions represent a small percentage (around 0.1%) of the total predictions integrated by Fisher in our work, and therefore with little influence on the overall increase in prediction power observed.

The number of STRING predictions is significantly smaller that the PG predictions indicating a much lower coverage for all the methods compared (Table 2).

	Yeast		Human	
Method	STRING	PG	STRING	PG
FisherW predictions	89,037	10,642,398	87,102	70,908,243
neighborhood	17,137	-	18,391	-
fusion	1,119	678,928 (cath)<br>336,781 (pfam)	3,943	32,259,881 (cath)<br>24,984,943 (pfam)
co-occurence	2,791	-	20,348	-
coexpression	72,049	10,371,735	49,382	26,292,126
hiPPI	-	12,070	-	86,099

Table 2. Comparison of the numbers of STRING and PG *ab-initio* predictions in yeast and human. First column: methods. Following columns: number of predictions retrieved by each *ab-initio* method independently for the STRING and PG data in yeast and human. CODAcath (cath) and CODApfam (pfam) number of predictions are also indicated.

Since a given method can predict large numbers of predictions without there being any functional information associated with the predictions, the total number of predictions is not a good indicator of the methods' performance in itself, but the number of accurate predictions above a significant precision level (e.g. 80% precision) is a useful measure.

Therefore, the STRING predictions were validated with the same Gossr and Int gold standard datasets used to validate the PG predictions in our work (Figure 4).

The STRING Fisher predictions' scores show correlation with precision in yeast and human (see Figures 4a and b, respectively). However, the STRING Fisher's prediction power is much lower than the PG predictions. Whilst, in yeast, the number of STRING predictions with precision ≥80% number 14,293 in Gossr validation and 19,849 in Int validations respectively (see Figure 4a) the comparable PG validations in yeast yielded 95,351 and 240,840 predictions above 80% precision (see Figures 1a in the main manuscript and 3a). In human validation, STRING showed worse performance than in yeast, probably due to the difficulties in implementing *ab-initio* prediction methods in upper eukaryotic organisms like human (Ranea et al., 2007 [50]). STRING human validation shows 100 predictions with precision ≥80% in Gossr validation, 550 and 650 predictions in the Int and Reactome_int validation respectively, while the comparable figures in the PG human validations are 1,052,579, 455,450, and 3,823,840 in the Gossr, Int and Reactome_int respectively with precision ≥80% (see Figures 4b and compare versus Figures 1b in the main manuscript and 3b). The integrated PG datasets clearly outperform the integrated STRING datasets and therefore constitute an accurate and comprehensive dataset of *ab-initio* predictions to analyse and compare with the KG datasets.

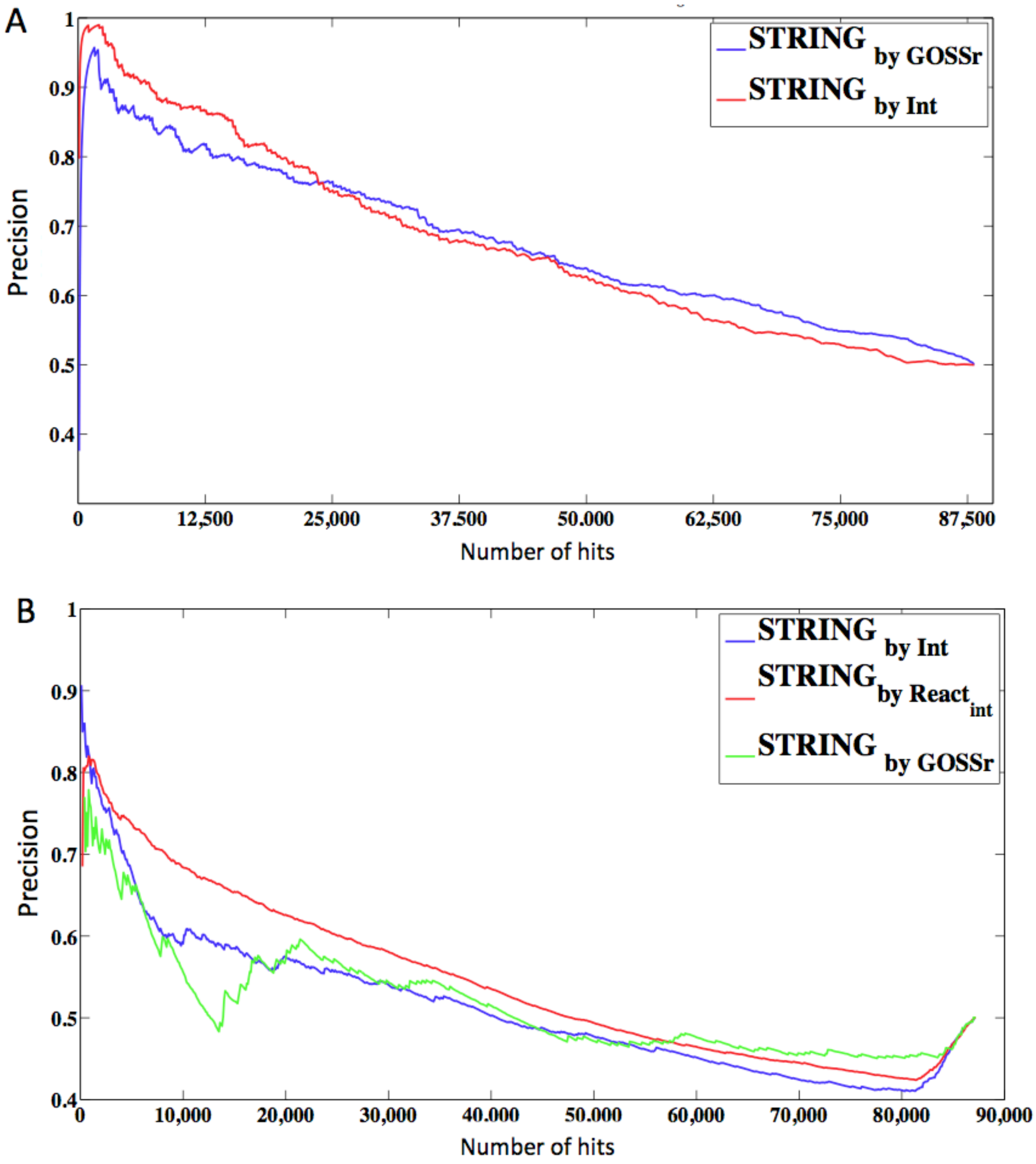

Figure 4. Validation of the FisherW integration of the STRING *ab-initio* prediction datasets in yeast and human. STRING_FisherW predictions were validated with Gossr and Int in yeast (A) and human (B), and with Reactome_int in human (B). Precision (y-axis) is represented versus number of predictions –Recall- (x-axis).

6. Assessment studies for the Bayes integration of CODA, hiPPI and GECO datasets.

In order to contrast the results of the Fisher weighted method with other methods, exploiting prior knowledge, we compared the Fisher weighted method to the Naïves-Bayes classifier, which employs a semi-learning approach. We used the Gossr as a True Positive (TP) training dataset and a randomisation of this dataset for the True Negative (TN) training dataset (with 1,000 iterations). These TP and TN datasets were useful for learning the corresponding parameters of our model and subsequently for calculating, by bayesian inference, the posterior maximum likelihood for every pair involved in the

integration. A Likelihood Ratio (LR) was calculated for every TP and TN integrated dataset's p-values as follow:

$LR(p_1,p_2,\ldots,p_n) = P(p_1,p_2,\ldots,p_n\,|I)/P(p_1,p_2,\ldots,p_n\,|{\sim}I) = \prod_{i=1}^{n} [P(p_i|I)/P(p_i|{\sim}I)]$

(In the $\prod_i$ LR calculation it is assumed the conditional independence of the integrated datasets).

And therefore, by inference of Bayes rule, we are interested in determining the posterior odds ratio of interaction between two proteins in our integration:

$O_{post} = P(I|p_1,p_2,\ldots,p_n\,)/P({\sim}I|p_1,p_2,\ldots,p_n) = P(I)/P({\sim}I) * P(p_1,p_2,\ldots,p_n\,|I)/P(p_1,p_2,\ldots,p_n\,|{\sim}I) = O_{prior} * LR(p_1,p_2,\ldots,p_n)$

Where O_{post} and O_{prior} are the posterior and prior odds ratios respectively and I is a binary variable for the interaction and absence of interaction in the case of ~I. Finally, we termed $p_1,p_2,\ldots,p_n$ as the scores of datasets to being integrated.

Bayes integration of the datasets from the four individual methods (GECO, CODAcath, CODApfam and hiPPI) was implemented as described above for yeast and human, and the Bayes integration was compared against the Fisher integration. The yeast and human Bayes integrations were validated and compared using Int and Reactome_int datasets, since the Gossr datsets could not be used for both, training and validating proposes.

Bayes integration produced uneven results in yeast and human compared to Fisher (Figure 5). Whilst Fisher outperforms Bayes for the highest levels of precision in yeast (see left side of the Figure 5a), in human Bayes performs better (see Figure 5b). These results show that the Fisher's integration gives a good performance compared to a trained method, despite the fact that this has the advantage of learning from the experimental (KG) information to predict PPIs. Therefore, despite the increase in precision achieved by using Bayes with the human data, Bayes, as with any trained and/or supervised integration method, could not be used in our analysis since we would then be comparing Bayes PG models, that were not completely independent from the KG training datasets, against the KG models.

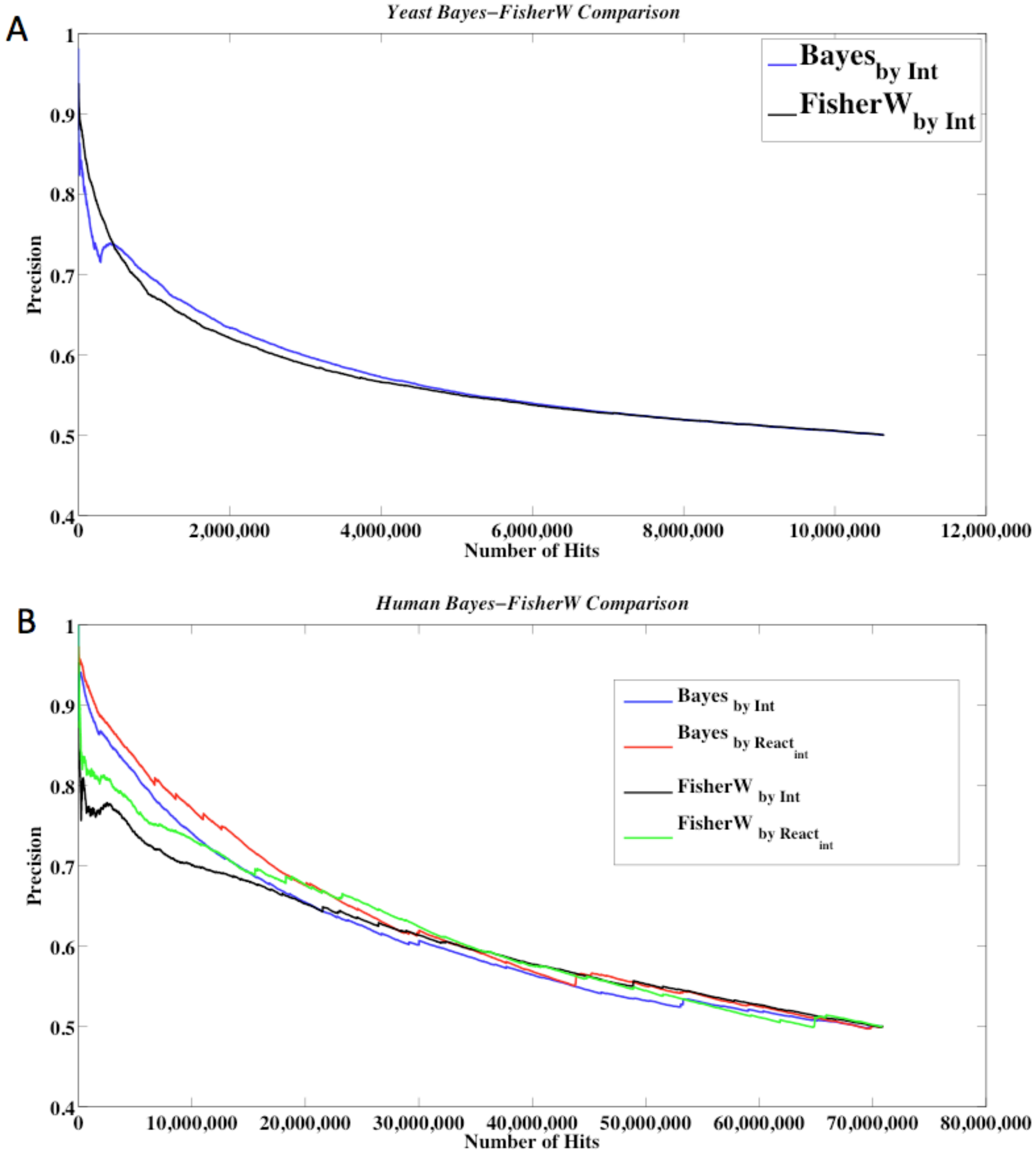

Figure 5. Comparison of the Fisher and Bayes integrations of the PG datasets in yeast (A) and human (B). Precision (y-axis) versus number of predicted hits –Recall- (x-axis).

7. Calculating topology features for the PG and KG protein networks.

The following topological parameters were calculated for the PG and KG networks:

Degree Statistic or Vertex Degree Connection, the most commonly calculated parameter. Measurements in classical undirected graphs indicate that the power-law growth described as $ck^{-\gamma}$ with $\gamma>0$ and $c>0$ and its associated exponents ($\gamma\approx2$-3 typically for stable models) are significant statistics for the classification of graphs and can be used to determine the similarity of the predicted network model to the real network model [36-40].

Degree Correlation or Assortativity [39,40]**,** this parameter measures the preferential attachment of a new node i.e. the likelihood that a new node will be associated with

others that are highly connected in the graph. Together with power-law growth this is a sufficient and necessary condition for Scale Free (SF) network models [37,38].

Clustering Distribution, this is a density function based on the probability of each node belonging to a completely connected triplet, and is a necessary condition for proving a Hierarchical or modular organization [41, 52].

Clustering Average Coefficient Statistic or the mean probability of finding triangles in the networks [37], which would support the scale free behaviour of our networks [38].

Distance Statistics, in our case this was calculated by solving the all pairs shortest-path problem (APSP) in polynomial time [42]. We have used some more 'compact' derived statistics as well, such as:

Average or Characteristic path length ℓ parameter [37], the arithmetic mean of all connected pair distances in the graph used for assessing small world properties [36,60]. For disconnected sub-graphs we set $\ell=\infty$.

Radius, Diameter and Eccentricity Statistics [42] By fixing one argument of the distance, the analysis of this eccentricity might be usefully reduced to $\varepsilon cc(n_i)$ as the maximal distance to another node in the graph. In our case this can be calculated from the distance matrix $O(n_i^2)$ (obtained from the *single-source shortest-paths problem* solution (SSSP) time). The radius of the Graph, Rad(G), as the minimal eccentricity of all vertices. The diameter of a graph G, diam(G), as the maximal distance between two arbitrary (connected) vertices (used in testing the network modularity [52]).

Algorithmic Aspects and Path Algebra, Our networks were all treated as cycles of non negative weight [32] so that the problem of determining distances could be solved in polynomial time and by well established algorithms provided as functions deployed in Matlab MathWorks, C++, Java, Octave and Python high-level languages. Assuming that G=(V,E) is a weighted undirected graph without cycles of negative weight the approach adopted for the calculation of the distance matrix is given by matrix multiplication over the path algebra [42].

Let $d_i(u, v)$ be the weight of a shortest path (i.e. a path of minimal weight) from u to v using at most i edges. This implies

$$d_0(u,v) = \begin{cases} 0 \\ \infty \end{cases} \textit{if } u=v, \textit{ otherwise } \infty.$$

Since a path with at most $i + 1$ edges either has at most i edges or consists of a path of length $\leq i$ to a predecessor v' of v and the edge (v', v), we have

$$d_{i+1}(u,v) = \min_{v' \in V}(d_i(u,v), d_i(u,v') + w(v',v)).$$

8. The set of PG models obtained using different cutoffs in yeast

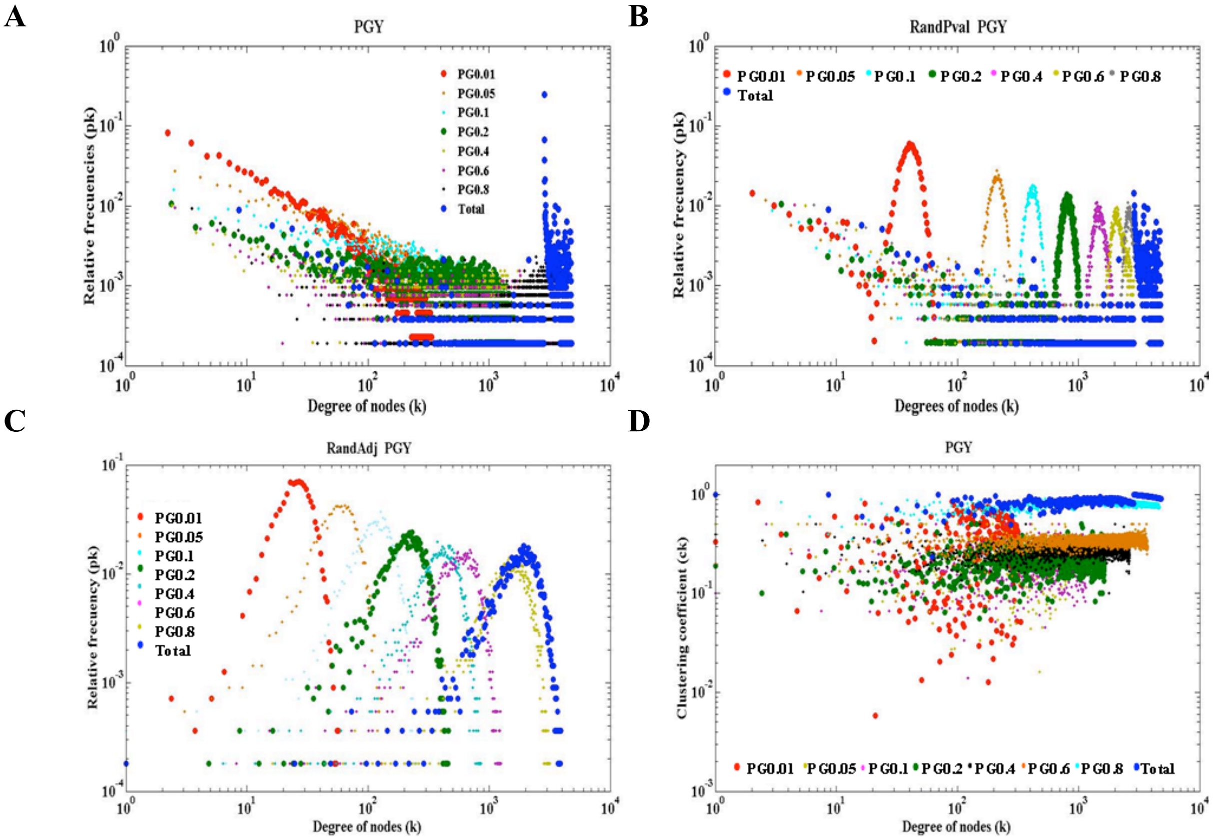

Figure 6. Pictures of the different PG Yeast network models presented in the main manuscript. A) the non-random network, B) and C) the p-value and adjacency random models respectively. Finally, D) the clustering coefficient distribution. The points referring to distributions depicted in the main manuscript have been enhanced in size.

Figure 6 presents the entire set of network models studied in the analysis including those introduced in the main manuscript obtained by every cutoff applied in the analysis. Notice panel D) presents the PG Clustering distribution in Yeast.

Note: There will be not a section for all KG models because the complete sets of results have already been presented in the main manuscript.

9. The set of KG models obtained by using different cutoffs in KG human

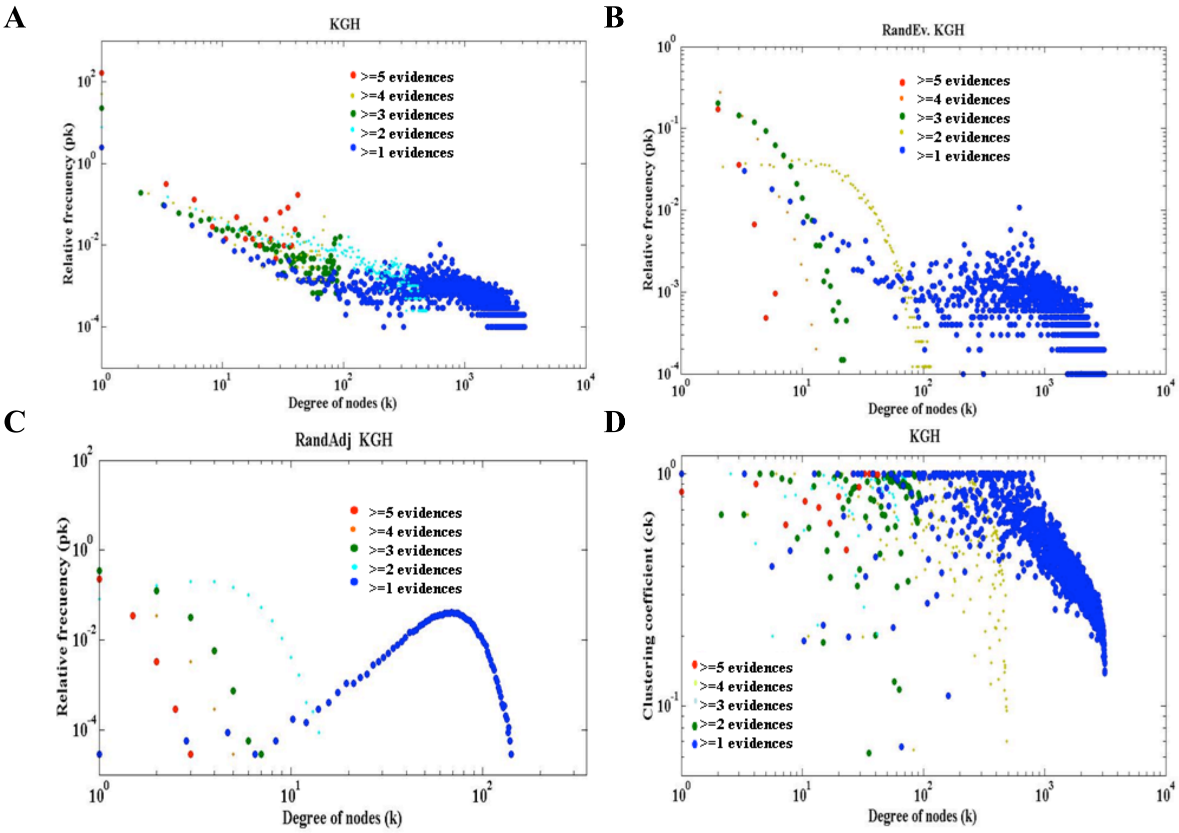

Figure 7. As for Figure 6 but showing data for the Human KG-grams. Similar trends are observed.

Figure 7 presents the entire set of network models studied in our analysis, including those shown in the main manuscript, and obtained using different cut-offs. Note: panel D) presents the PG Clustering distribution in Human.

10. The set of PG models obtained by using different cut-offs in PG human

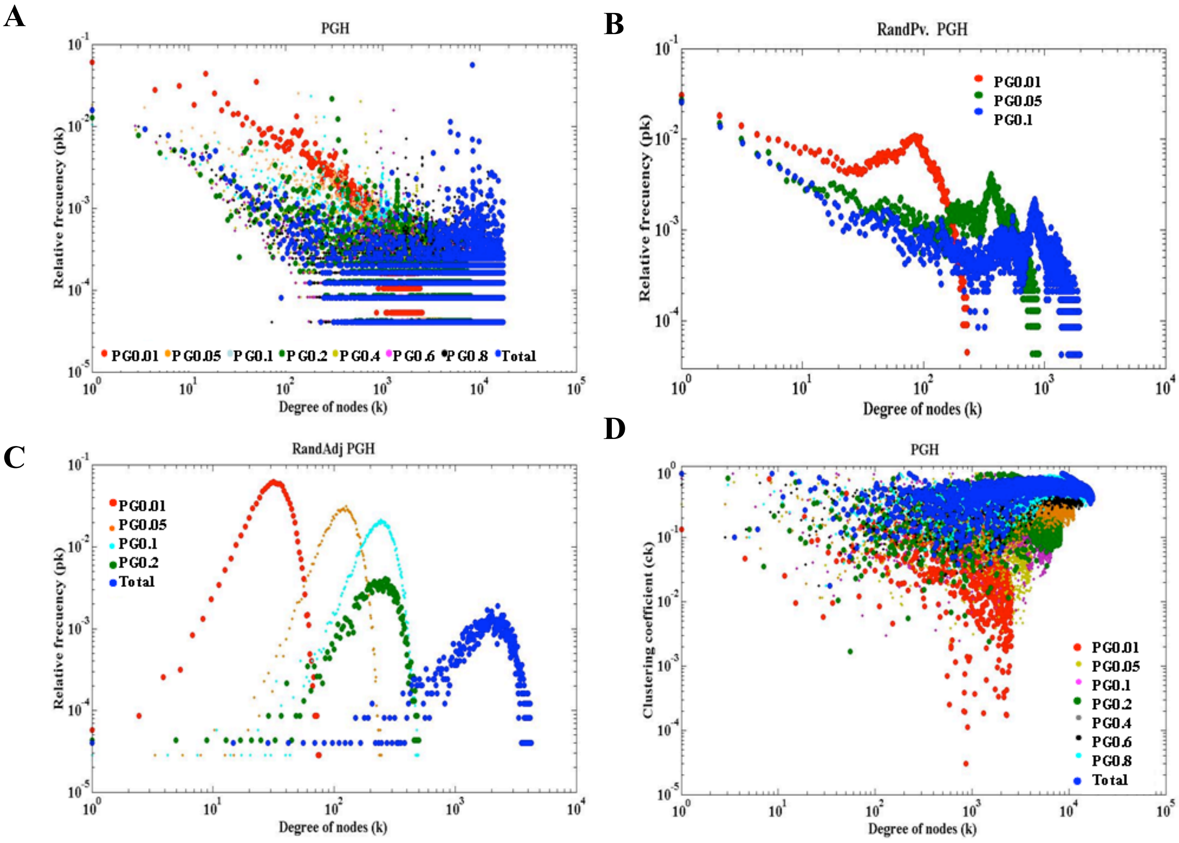

Figure 8. As in Figure 6 but for the Human PG-grams. The same trends are observed again. A scale-free character is observed, typical of the real-networks, as well as a non-hierarchical organization.

11. Assortativity of yeast and human networks

A

B

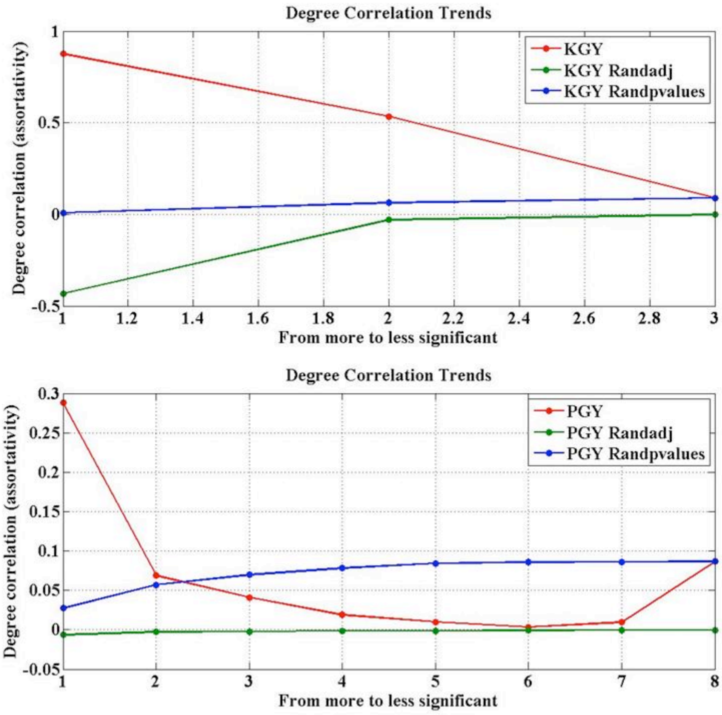

Figure 9. This shows the degree correlation trends for the different Yeast KG and PG networks presented in this paper.

A

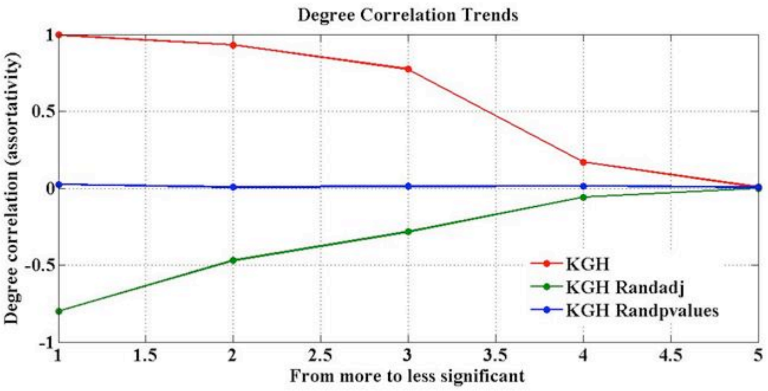

B

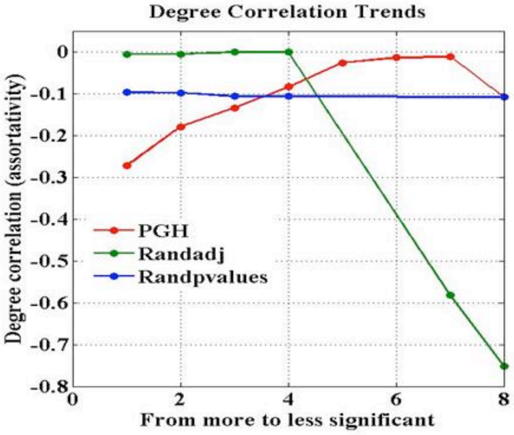

Figure 10. These results provide a view of the degree correlation between the different networks presented in this paper. The final plot explains why there is such a radical decay of the degree distribution in the human PG-gram in Figure 3b (main manuscript). Notice the contrasting behaviour of the assortativity in human PG compared to the behaviour in yeast Figure 2b.

Assortativity trends in Figures 9 and 10 are in agreement with the results shown in Figures 2 a-b and 3 a-b of the main manuscript in which the Degree distribution (k_i) remains linear for edges with strong statistical weight and the Gaussian random effect appears when those edges have weaker statistical weight.

12. Hierarchical organization

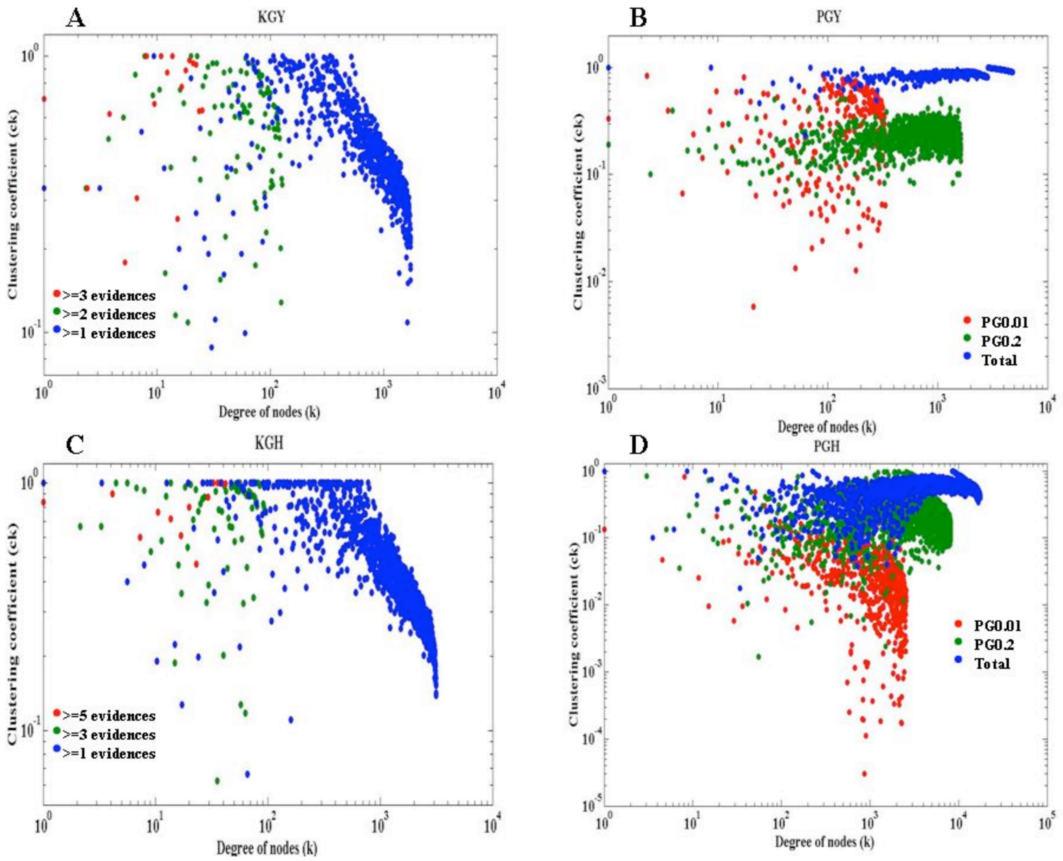

Figure 11. Clustering distributions in yeast (panels A and B) and human (panels C and D) PG and KG networks respectively. The legend for panels B, D indicates the level of significance selected in yeast and human PG networks (e.g., $PG_{0.01}$ = the PG network with p-value threshold of ≤0.01). Axes are the clustering coefficient (y-axis) versus connectivity (ki) per node (x-axis).

13. Other Parameters Involved in the Analysis of the Networks

In this section we present the parameters measured in the analysis of the networks.

**KGYeast significance levels**	**Density**	**Cluster average**	**Characteristic Path Length (ℓ)**	**Radius**	**Diameter**
**>=3 evidences**	**6.6789*10^-5**	**0.03537**	**4.9780**	**2.000**	**16.0000**
**>=2 evidences**	**0.0015**	**0.25814**	**4.4867**	**2.000**	**11.0000**
**>=1 evidences**	**0.0437**	**0.48762**	**2.2712**	**4.000**	**6.0000**
**KGHuman significance levels**	**Density**	**Cluster average**	**Characteristic Path Length (ℓ)**	**Radius**	**Diameter**
**>=5 evidences**	**2.1809*10^-06**	**0.00337**	**2.5480**	**1.0000**	**10.0000**
**>=4 evidences**	**8.7124*10^-06**	**0.01173**	**2.3100**	**1.0000**	**9.0000**
**>=3 evidences**	**2.0545*10^-05**	-	-	-	-
**>=2 evidences**	**0.000109**	-	-	-	-
**>=1 evidences**	**0.002914**	**0.15261**	**1.0100**	**1.0000**	**2.0000**

Table 3. Network parameters used in the analysis: density, cluster average, characteristic path length average, radius and diameter (see section 6). The rows are the KG networks for yeast and human species.

PGYeast significance levels	Density	Cluster average	Characteristic Path Length (ℓ)	Radius	Diameter
≤0.01 pvalues	0.0016	0.1961	3.2137	2.0000	9.0000
≤0.05 pvalues	0.0169	0.1584	2.5876	1.0000	12.0000
≤0.1 pvalues	0.0345	0.1819	2.2037	1.0000	10.0000
≤0.2 pvalues	0.0648	0.2085	1.9237	1.0000	9.0000
≤0.4 pvalues	0.1361	0.2541	1.7099	1.0000	7.0000
≤0.6 pvalues	0.2035	0.3061	1.5441	1.0000	7.0000
≤0.8 pvalues	0.5462	0.7498	1.3411	1.0000	3.0000
Total	0.6822	0.8960	1.2041	1.0000	2.0000
PGHuman significance levels	Density	Cluster average	Characteristic Path Length (ℓ)	Radius	Diameter
≤0.01 pvalues	0.0012	0.0424	2.6974	1.0000	12.0000
≤0.05 pvalues	0.0053	0.1054	2.6641	1.0000	12.0000
≤0.1 pvalues	0.0118	0.1353	2.5138	1.0000	11.0000
≤0.2 pvalues	0.0215	0.1710	-	-	-
≤0.4 pvalues	0.0454	0.2271	-	-	-
≤0.6 pvalues	0.0694	0.2987	-	-	-
≤0.8 pvalues	0.0907	0.3651	-	-	-
Total	0.1158	0.4252	1.6035	1.0000	5.0000

Table 4. Network parameters: density, cluster average, characteristic path length average, radius and diameter (see section 6). The rows are the PG networks for yeast and human species.

In Tables 3 and 4 several measures are shown which support the highly modular nature of our networks: as the significance levels of the PG and KG models increases there is a decreasing density with concomitant decrease in the cluster average. This correlation between density, cluster average and significance level together with the fact that the radius remains almost equal and both diameter and ℓ increase appreciably, is consistent with the other topological signals of high modularity found in our modelled networks. These include: relatively low assortativity (in human lower than in yeast), scale free exponents below 3, and no hierarchical structure (detailed support of these statements can be found in references [37, 39, 42]). Note that the density parameter was defined as the probability of obtaining triangles in any network, and that radius, ℓ, and diameter measurements are referred to the largest component of a network in accordance with definition of path algebra introduced in section 7.

Some of the parameters are not explored in human because of the high computational cost in calculating these values. Nevertheless, the tendency for high modularity is confirmed in the other parameters calculated for human.

To further illustrate the modular behaviour of our models we present two models (A – for human and B –for yeast in Figure 12) highlighting the difference in modularity of the yeast and human networks. Notice a higher assortativity in the yeast network (as in most real-life networks) compared to the human example. These examples support the proposed possible configuration of our networks, although they do not necessarily depict an exact model of them.

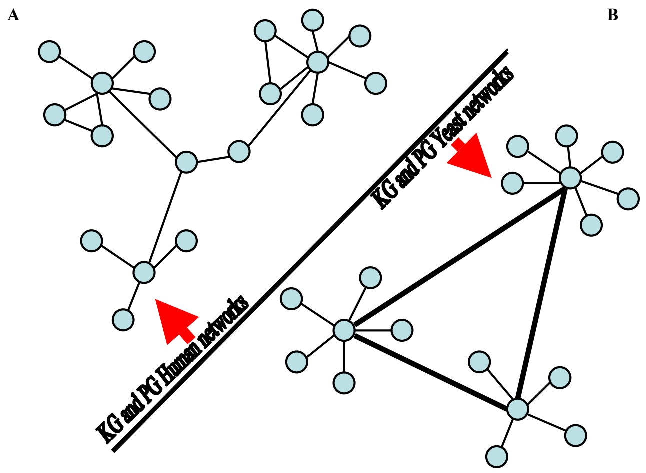

Figure 12. Two disassortative networks. (A) High-degree nodes are loosely interconnected in the case of human KG and PG networks. (B) High-degree nodes are tightly interconnected in yeast KG and PG networks.

14. The GO semantic similarity refined dataset (Gossr) used for validating the prediction methods.

In order to increase the quality and reliability of the human and yeast validation datasets, those annotations with term evidence type Inferred from Electronic Annotation (IEA), No biological Data available (ND) and those inferred from Computational Analysis were removed. We expected the source data of CODA, GECO and hiPPI to have overlap with IGC (Inferred from Genomic Context), IEP (Inferred from Expression Pattern) and IPI (Inferred from Physical Interaction) annotation sets respectively. To minimise this overlap and prevent circularity, these evidence codes (IGC, IEP and IPI) were removed.

GO annotations of human and yeast proteomes were obtained from UniProt GOA proteome sets 17th-October-2008 (Human file: 25.H_sapiens.goa; yeast: 40.S cerevisiae.ATCC 204508.goa with whole proteomes coverage by GO annotation of 95.5% and 72.5% respectively) downloaded from the Gene Ontology Annotation (GOA) database located at the European Bioinformatics Institute (Hinxton - http://www.ebi.ac.uk/GOA/).

15. Quantitative comparison of intersections and unions of KG and PG

Intersection, Fusion and Source Networks	# Edges	# Nodes
**KGY U PG0.01Y**	760,057	5,366
**KGH U PG0.014H**	2,821,556	24,056
**KGY**	682,079	5,261
**PG0.01Y**	95,351	4,374
**KGH**	1,783,025	10,095
**PG0.014H**	1,052,579	19,618
**PG0.01Y \ KGY ∩ PG0.01Y**	77,978	105
**PG0.014H \ KGH ∩ PG0.014H**	1,038,531	13,961
**Yeast (total proteome)**	-	5586
**Human (total proteome)**	-	34912

Table 5. Quantitative analysis (in terms of the edges and real nodes) of the union and intersection of the source sets (Yeast (Y) and Human (H)). The total number of proteins (nodes) in the yeast and human proteomes are also indicated at the bottom of the Table.

16. Functional enrichment analysis of the yeast and human dark (hidden) hubs.

We have performed a comparative enrichment analysis of the top 10% and bottom 10% of the PGk_i_er ranked lists, against the human proteome background datasets, using the DAVID algorithm [43]. DAVID includes functional annotation from an extensive number of public resources and identifies enriched biological themes, particularly GO terms, computing a p-value for the observed enrichment. Results indicate that the dark hubs in our dataset (top 10% of the PGk_i_er ranked lists) are significantly enriched in proteins integral to membrane in yeast (Table 6), compared to the bottom 10%, and in unknown (i.e. with no functional annotation) proteins in yeast and human (Table 7).

	GO term	GO cat.	Top 10 %	Bot. 10 %	Tot. %	P-val	GO: number Ids	GO definition
Yeast	Intrinsic to membrane	cc	111		23	9,10 E-06	0031224	Located in a membrane such that some covalently attached portion of the gene product, for example part of a peptide sequence or some other covalently attached moiety such as a GPI anchor, spans or is embedded in one or both leaflets of the membrane.
	Integral to membrane	cc	106		22	1,80 E-05	0016021	Penetrating at least one phospholipid bilayer of a membrane. May also refer to the state of being buried in the bilayer with no exposure outside the bilayer. When used to describe a protein, indicates that all or part of the peptide sequence is embedded in the membrane.
	Regulation of transcription	bp		110	21	1,20 E-08	0045449	Any process that modulates the frequency, rate or extent of the synthesis of either RNA on a template of DNA or DNA on a template of RNA.
	Non-membrane-bounded organelle	cc		132	25	2,60 E-03	0043228	Organized structure of distinctive morphology and function, not bounded by a lipid bilayer membrane. Includes ribosomes, the cytoskeleton and chromosomes.
	Intracellular non-membrane-bounded organelle	cc		132	25	2,60 E-03	0043232	Organized structure of distinctive morphology and function, not bounded by a lipid bilayer membrane and occurring within the cell. Includes ribosomes, the cytoskeleton and chromosomes.
human	Extracellular region	cc		423	21	2,00 E-24	0005576	The space external to the outermost structure of a cell. For cells without external protective or external encapsulating structures this refers to space outside of the plasma membrane. This term covers the host cell environment outside an intracellular parasite

Table 6. Gene-annotation enrichment analysis of the yeast and human dark (hidden) hubs. Gene-annotation enrichments in GO of the 10% top of hubs and the bottom 10% of nodes in the yeast and human protein lists, ranked by their PGk_i_er values, performed using the DAVID algorithm with the human proteome as the background (Dennis et al., 2003; http://david.abcc.ncifcrf.gov/; see in Results the section: "Analysing the 'dark matter' in the PG models." and in Methods the section: "Calculating the PGk_i enrichment ratio and the PG functional enrichment"). PGk_i_er values rank the dark (hidden) hubs at the top of the list. These are proteins with high connectivity in the PG model and low connectivity in the KG models; while those hubs with few connections in the PG models compared to the KG models are ranked on the bottom. The Table shows from left column to right: specie, yeast and human; name of the GO term; GO category: cc (cellular component) and bp (biological process); Top 10% - results related to the enrichment analysis of the top 10% of the ranked lists; Bottom 10% - results related to the bottom 10% of the ranked lists; Tot. % - protein enrichment percentage over the analysed datasets; p-value, statistical significance provided by DAVID – note that all the enrichment data shown are statistically highly significant; GO identification number; and functional definition in GO. Only the Tot.% enrichment values equal or greater than 20% are reported. Enrichment results show that 22% of the top 10% dataset proteins are "Integral to membrane" in yeast, while 25% of the bottom 10% dataset proteins are annotated as "Intracellular non-membrane-bounded organelle". In human there is no significant enrichment equal to or above 20% in the top 10% dataset, while in the bottom 10% of the dataset 21% of proteins are annotated with the "Extracellular region" GO localization term. The results in yeast statistically support the observation that the hidden hubs are significantly related to membrane proteins, which are difficult to isolate by conventional purification protocols.

	yeast		human	
	**Top 10%**	**Bottom 10%**	**Top 10%**	**Bottom 10%**
**Known**	475	522	1907	2125
**Unknown**	61	14	286	68
**Total**	536	536	2193	2193

Table 7. Number of known and unknown proteins in the yeast and human dark (hidden) hubs datasets. This table shows the number of proteins with functional annotation (known) and without functional annotation (unknown) found by DAVID in the top and bottom 10% of the protein lists ranked by their PGk$_i$_er values in yeast and human. The ratio of unknown proteins in yeast (61/14) and in human (268/68) are both about 4 times higher in the top 10% than in the bottom 10% of the dataset, indicating that the dark (hidden) hubs datasets (top 10%) are enriched in proteins without any current functional knowledge.

17. Context analysis and validation

The following example represents the typical context analysis framework:

	1	2	3	4	5	6	7
**Prot. 1**	0	0	0	1	0	1	1
**Prot. 2**	0	0	0	1	0	1	1
**Prot. 3**	1	1	0	0	1	0	0
**Prot. 4**	1	1	0	0	1	0	0
**…**	…						

Figure 13. Context matrix of interactors. This matrix represents the framework of second order or context prediction analysis. Prot.1 and Prot.2 share the same partners as do Prot.3 and Prot.4 but there are no binary links between proteins in these pairs.

We observe in Figure 13, for example, that in the comparison between Prot.1 and Prot.2 only three matching bits (1-1) are found, whilst between Prot. 1 and Prot. 3 there is an absence of matching bits and between Prot. 3 and Prot. 4 two matching bits (1-1) are found and one non matching bit (1-0). It can be seen as well that there is not a direct relationship between Prot. 1 and 2 or between Prot. 3 and 4, consequently the prediction retrieved using context could not be detected from their primary interactions.

18. Functional association predictions based on context information in the PG networks

The protein association profiles (i.e. vectors of interacting proteins for each protein in the PG networks; see Methods) were compared between all protein pairs in order to retrieve additional association signals not explicitly present in the first order predictions. Similarities between protein association profiles were calculated using three different measurements: bits and specific bits for human and yeast (see methods), and congruence only for yeast [53] (congruence measure involves a combinatorial calculation which is not feasible given the large size of the human PG network; see Methods).

Bits and specific bits scores show very similar behaviour in all the KG datasets. In yeast specific bits slightly outperforms bits score for all the datasets, while in human the opposite is observed and bits slightly outperforms specific bits score except for Reactome_Int where bits calculation shows remarkable improvement over specific bits predictions (Figures 16e, 17e and 18e). However, the congruence measure in the yeast $PG_{0.001}$ network shows a poor performance close to random behaviour for practically all KG datasets in yeast (Figure 4a; and Figures 14 and 15 in Text S1). Therefore, we decided not to use congruence further as a measure.

The large sizes of both PG matrices (4,374 x 4,374 nodes in yeast and 19,618 x 19,618 nodes in human; see Table 5), make it very unlikely that two proteins would share a significant number of interactions in their respective association profiles by chance, which would explain the good performance of the bits score.

Similarity score performances vary according to the nature of the gold standard datasets used to validate them (Figure 20). If we consider the bits specific scores as the most stable measure, we observe that for yeast the second order approach is better at predicting protein relationships in KEGG, than predicting physical interactions (Int dataset) or ontological associations in the GO or FunCat databases (Goss and Foss datasets) (see Figure 20a and b). Whilst, in human, second order predictions seem to work much better at predicting associations in biological pathways (Reactome and KEGG) than predicting physical interactions (Int and Reactome_Int) and are even worse at predicting ontological relationships (GOSS and FOSS; see Figure 20c and d). These differences can probably be explained by the difference in noise (error) in the different biological sources used as gold standards to validate performance.

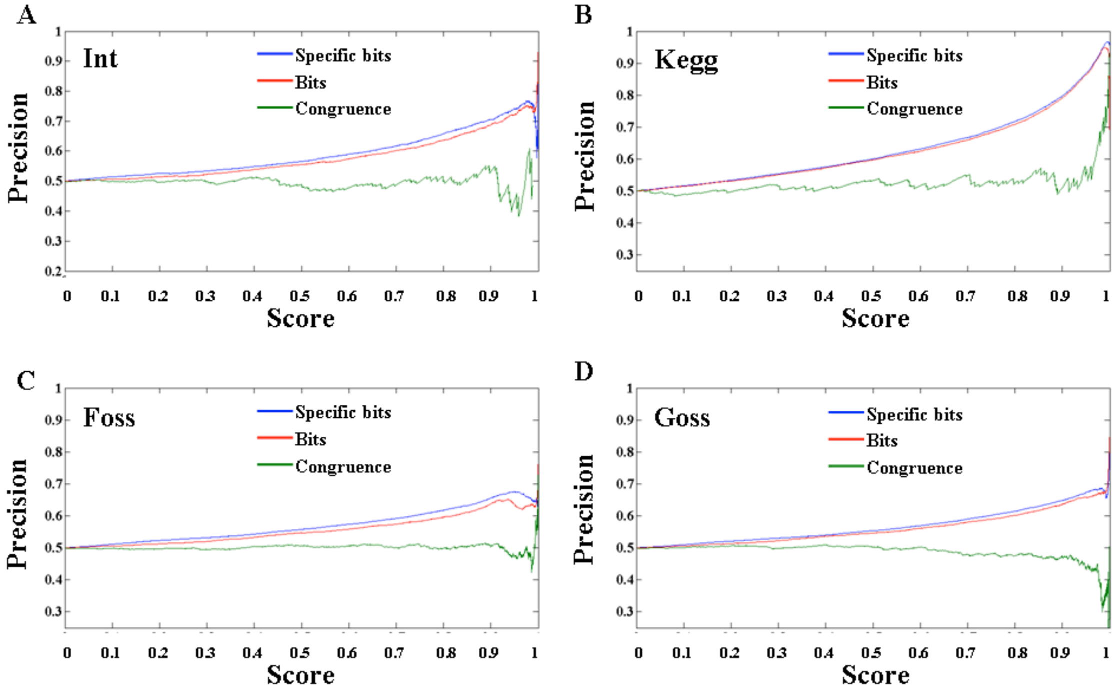

Figure 14. Yeast PG network context similarity measures validation with different gold standard datasets. The panels present the results for yeast in terms of precision versus score (specific bits, bits and congruence) in the context analysis. Panel A is the validation with the Int database as gold standard. Panel B validation for Kegg, C and D are validation with Foss (Funcat Semantic Similarity) and Goss (Semantic Similarity in GO annotations) respectively. In every panel a normalised comparison amongst the specific bits, bits and congruence context methods is shown.

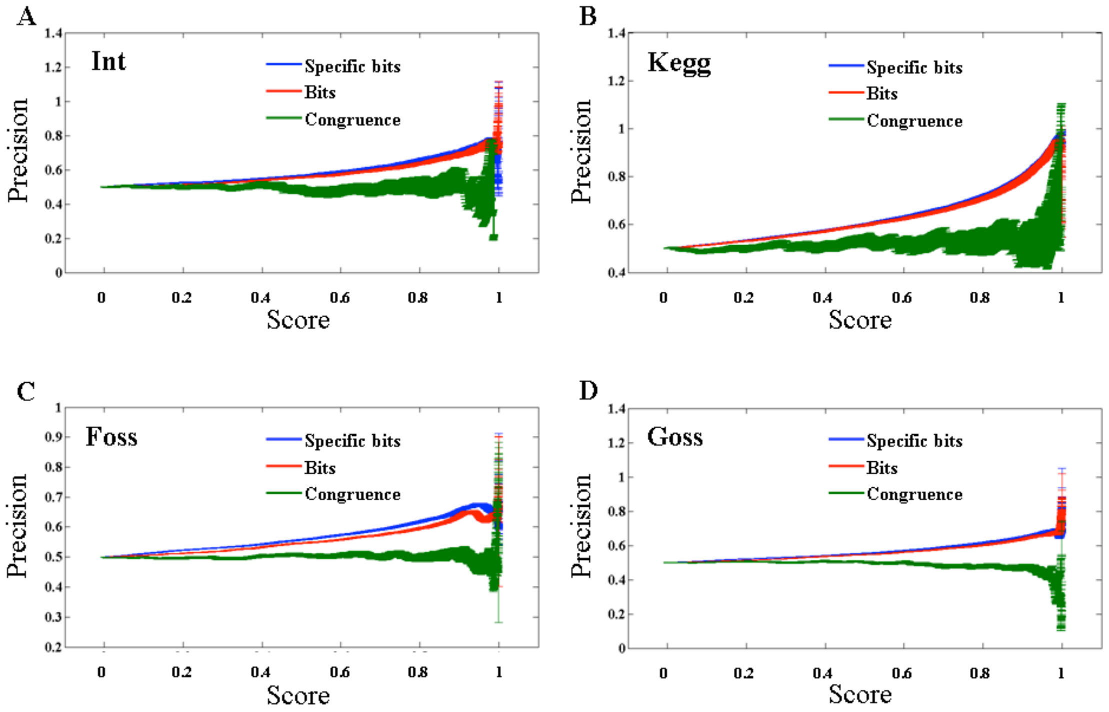

Figure 15. Yeast PG network context validation with different gold standard datasets and error bars indicated. These panels are the same of the Figure 11, but with error bars calculated for average points. Values of precision under a standard deviation of 1/3 of the mean were ignored.

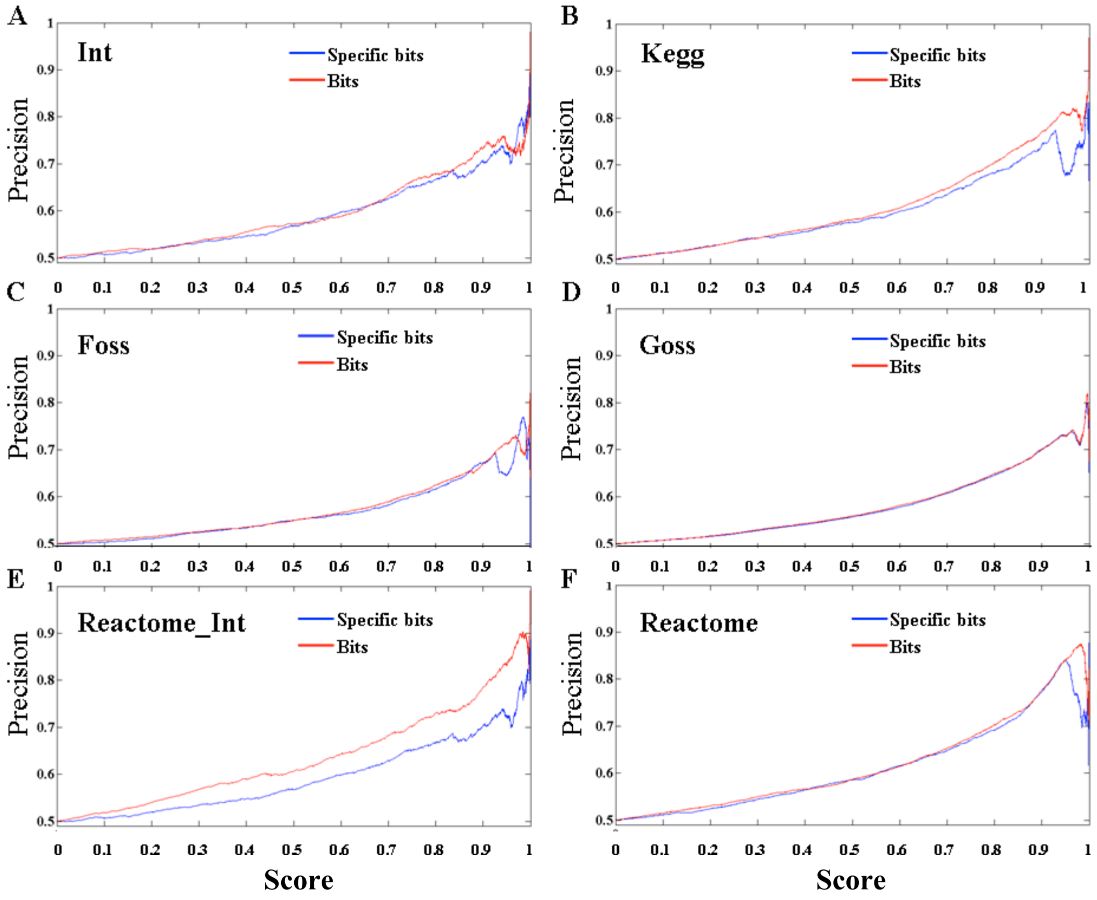

Figure 16. Network context validation of the human PG with different gold standard datasets. The panels present the results in the context analysis, for human, in terms of precision versus score (specific bits and bits). Panel A is the validation with the Int database as Golden Standard. Panel B validation for Kegg, C and D are validation with Foss (Funcat Semantic Similarity) and Goss (Semantic Similarity in Go annotations) respectively. Finally panels E and F present the validation using Reactome_int and Reactome as Gold standard. In every panel a normalised comparison amongst the specific bits and bits context methods is shown.

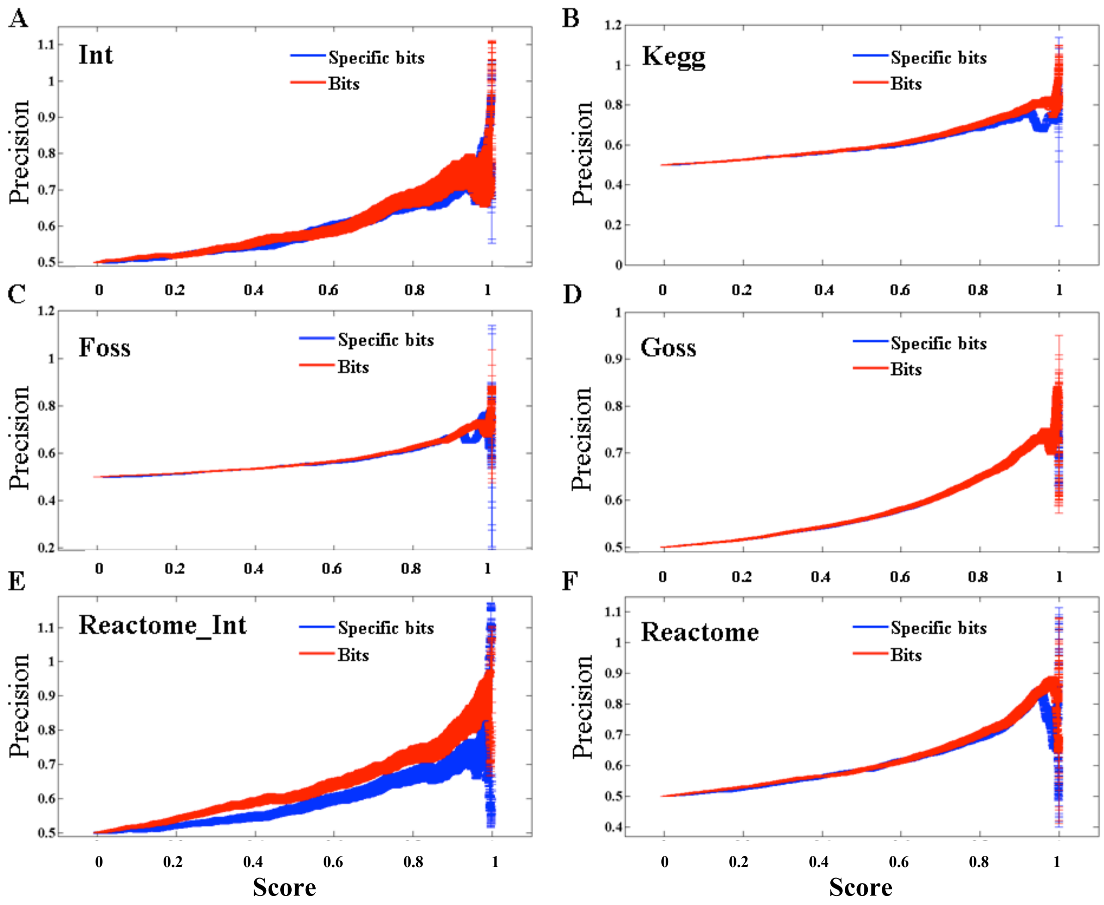

Figure 17. Human PG network context validation with different gold standard datasets and error bars indicated. These panels are the same of the Figure 11, but with error bars calculated for average points. Those values of precision under a standard deviation of 1/3 of the mean were ignored.

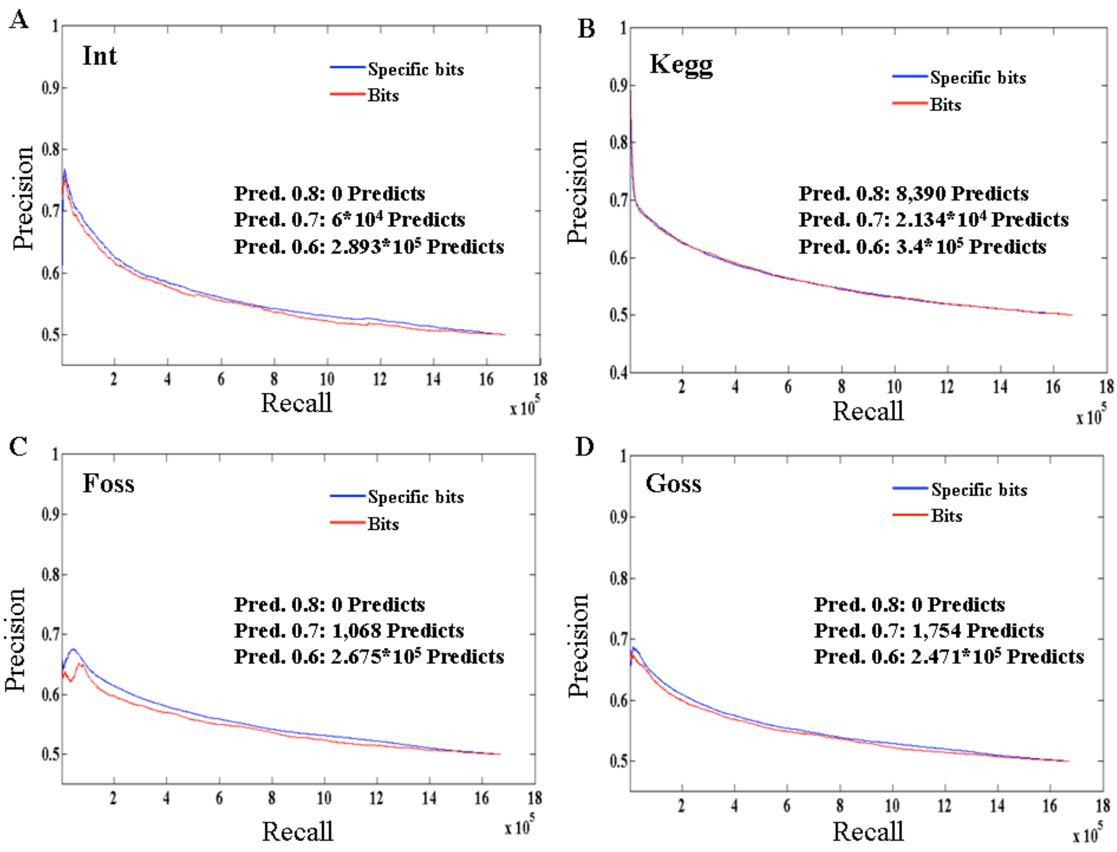

Figure 18. Validation of the yeast PG network prediction power with different gold standard datasets. The upper panels present the results for yeast in terms of precision versus recall as introduced in the main manuscript (specific bits and bits). Panel A is the validation with the Int database as gold standard. Panel B is the validation for Kegg, C and D are the validation with Foss (Funcat Semantic Similarity) and Goss (Semantic Similarity in GO annotations) respectively.

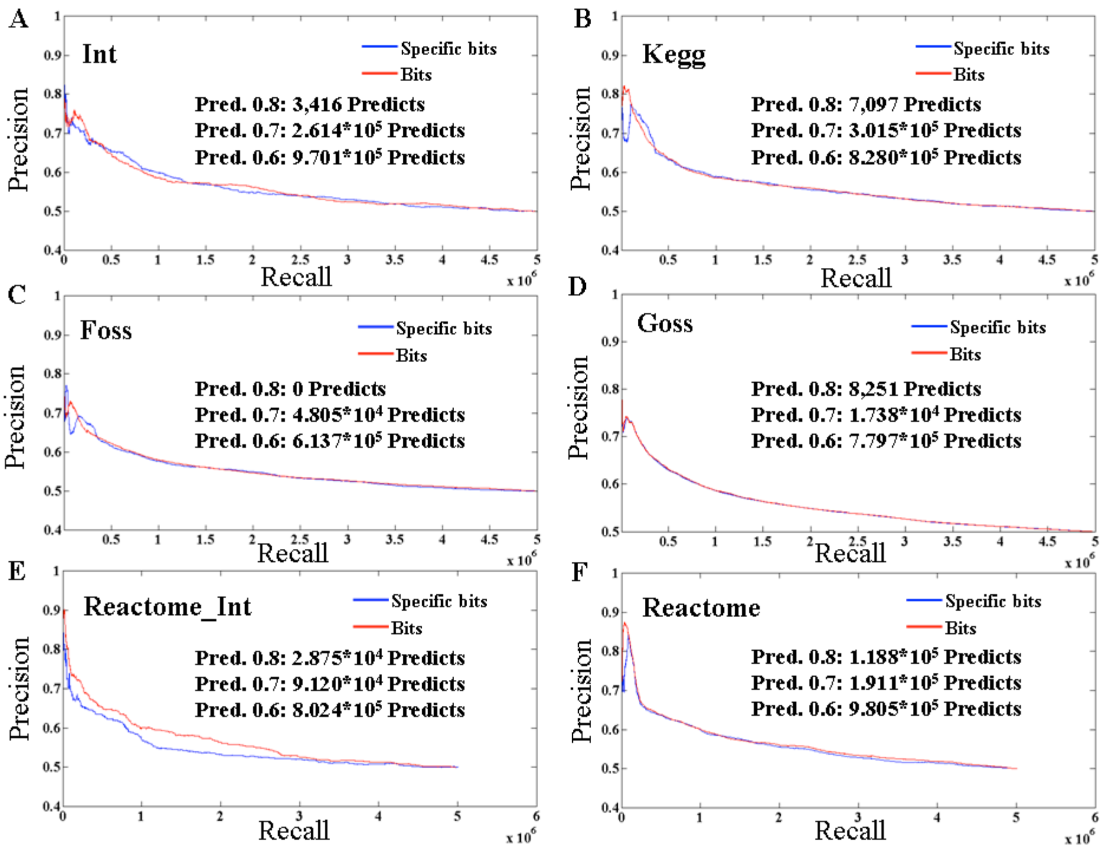

Figure 19. Validation of the human PG network prediction power with different gold standard datasets. These panels show the results for human in terms of precision versus recall as described in the main manuscript (specific bits and bits). Panel A is the validation with the Int database as gold standard. Panel B is the validation for Kegg, C and D are the validation with Foss (Funcat Semantic Similarity) and Goss (Semantic Similarity in GO annotations) respectively.

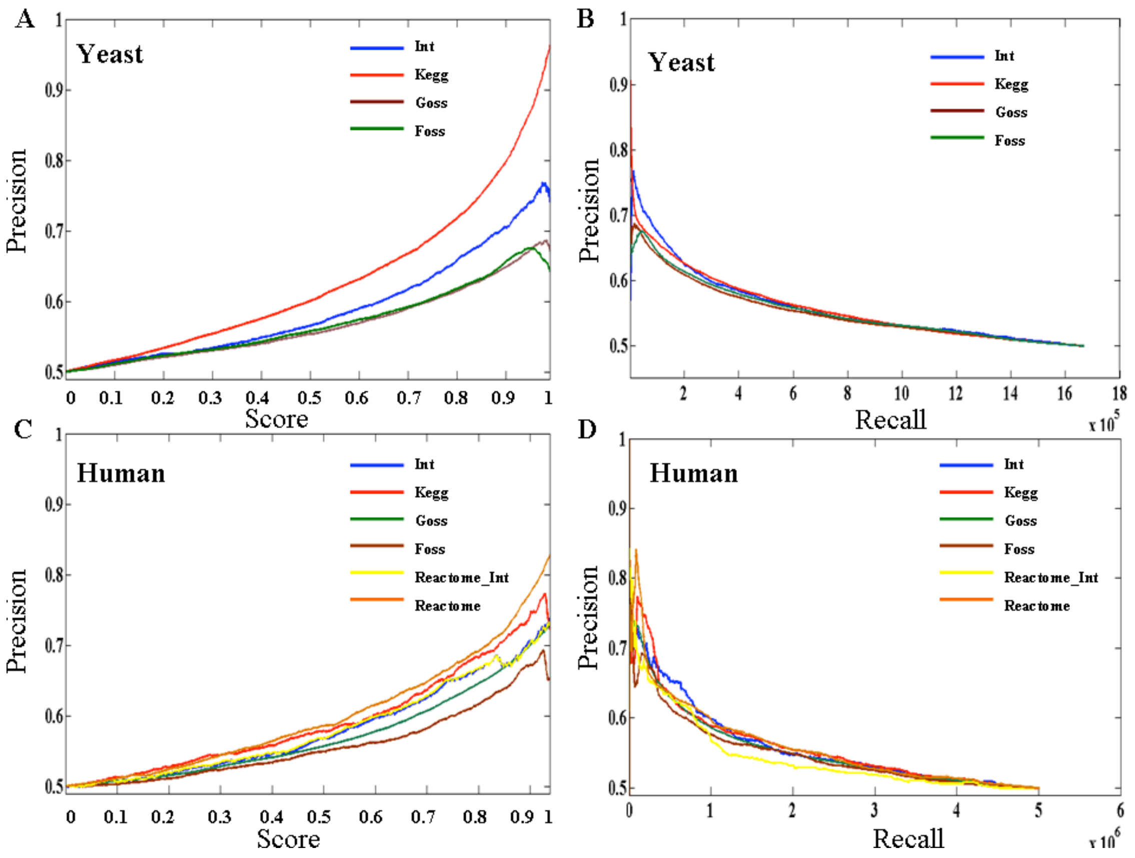

Figure 20. Validation of the PG networks functional context with different gold standard datasets. These plots present the precision value (y-axis) versus specific bits similarity score between protein pairs interaction profiles (x-axis in plots A and C) and versus Recall (# of pairs predicted, x-axis in plots B and D) for different sets of gold standards in yeast (plots A and B) and human (plots C and D) PG $_{0.001\&0.0014}$ networks. The aim of these plots is to use a common framework for showing the validations of all the PG networks derived by functional context. Gold standard datasets: Int, Kegg, Goss, Foss, Reactome-Int and Reactome are described in Methods.

19. Running the PG methods on the human and yeast proteomes

Proteome files were downloaded from the Integr8 [61] database June 2007. The Integr8 web portal (European Bioinformatics Institute –EBI-) provides easy access to integrated information about completed genomes and their corresponding proteomes. Available data includes DNA sequences (from databases including the EMBL Nucleotide Sequence Database, Genome Reviews, and Ensembl); protein sequences (from databases including the UniProt Knowledgebase and IPI) (http://www.ebi.ac.uk/integr8/EBI-Integr8-HomePage.do).

20. Weights in Fisher's integration statistic

As described in the main manuscript (Methods), Ztests were performed using Matlab to ensure that probability density function (PDF) distributions fit random Gaussian distributions (null hypthesis) at a 5% significance level. Calculation of weights in the Fisher's integration is based on running the *Monte Carlo and ESA algorithms* simultaneously. Both these approaches are well defined and well known in the field of

data integration. More specifically, the Monte Carlo simulation methods are particularly useful in studying systems with a large number of coupled degrees of freedom such as the data analysed in the main manuscript. We apply them as in a predictor-corrector system in which we predict a weight for our datasets and this is corrected at the same iteration in order to avoid a local optima effect. We summarize these methods in the following paragraphs:

The **Monte Carlo method** (Matlab package) provides approximate solutions to a variety of mathematical problems by performing statistical sampling experiments on a computer generating suitable random numbers, and observing that fraction of the numbers obeying some property or properties. The method is useful for obtaining numerical solutions to problems which are too complicated to solve analytically.

The method applies to problems with no probabilistic content as well as to those with inherent probabilistic structure. Among all numerical methods that rely on N − point evaluations in M − dimensional space to produce an approximate solution, the Monte Carlo method has absolute error of estimate that decreases as N superscript -1/2 whereas, in the absence of exploitable special structure all others have errors that decrease as N superscript −1/M at best. This may produce incorrect results, but with bounded error probability.

ESA Algorithm or Simulated Annealing algorithm is one of the best-known local search algorithms derived from thermodynamic principles. In the article **N. Metropolis et al.,** Equation of state calculations by fast computing machines, Journal of Chemical Physics 21 (1955), pp.1087-1092 [48] an algorithm simulating the behaviour of a system at a given temperature is proposed. At each iteration, a neighbouring solution, sol′ of the current solution, sol, is randomly generated. If the neighbour is better than the current solution, it is always accepted and becomes the current solution for the next iteration. Otherwise, the neighbour is accepted with a probability P_{accept} depending on the energy difference Δ between the two solutions (i.e. energy of neighbour minus energy of current solution) and a parameter t called temperature. This probability increases when the temperature increases and decreases when the energy difference increases. This selection scheme is called Metropolis criterion. The advantage of this scheme over Stochastic Hill Climbing (i.e. only accept better solutions) is the possibility to escape local optima. The short pseudo-code, the following text, the framework of a Metropolis chain of length L in temperature t is shown.

Metropolis chain

Algorithm Metropolis chain (initial, L, t)

```
{
  sol = initial
 repeat L times
   {
     sol′ = new neighbor of sol
     Δ = ENERGY(sol′ ) - ENERGY(sol)
    if Δ ≤ 0 then Paccept =1 else Paccept =exp(−Δ/t)
    if random(0,1)< Paccept then sol = sol′
   }
  return sol
```

}

Simulated annealing consists of a series of Metropolis chains at different decreasing temperatures. The aim of each Metropolis chain is to permit the system to reach thermal equilibrium. A slow cooling leads eventually to a frozen system yielding a good final solution.

In order to use simulated annealing algorithm for a specific optimization problem, an appropriate state space corresponding to the possible feasible solutions, a neighbourhood relation between the states, a cost function of each state and an appropriate cooling schedule should be selected. The role of neighborhood-relation is to express the similarity between the elements of the state space. The neighborhood of a state is typically defined as the set of the states that can be obtained by making some kind of local modifications on the current state.

Given the source node w, the state space of the simulated annealing is the set of all possible spanning trees rooted at the source node w and the cost of a state is the power cost of it. During the execution, the temperature decreases exponentially between successive Metropolis chains, i.e. the temperature ti after ith Metropolis chain is given by

$$t_i = \alpha \cdot t_{i-1} ,\ t_0 = \text{const},$$

where α is the so called cooling factor, which is a number close to 1 and t0 is initial temperature. The following idea is used to find a suitable neighbourhood structure.

21. Predicition validation, network topology and context analysis of the PG models without the hiPPI predictions.

Whilst CODA and GECO do not use any publicly available PPI information, hiPPI uses available experimental data by exploiting sequence similarity information between known interacting partners and their homologues to predict new interacting pairs (see section 1 in this text S1 document).

Therefore, there is a reasonable concern that the sequences we are predicting in human and yeast could be very close homologues of the sequences in the KG datasets and perhaps the weight accorded to the hiPPI predictions in the integrated prediction set (CODAcath, CODApfam, GECO and hiPPI) could be high enough to significantly bias the Fisher's integrated PG model so that the characteristics resemble those of the experimental KG network. Addressing this possibility we have repeated the main analyses of this work excluding the hiPPI predictions.

When hiPPI predictions were excluded we observed that the Fisher integration of the remaining predictions gives a similar performance predicting almost the same number of true PPIs in yeast and in human above 80% precision (see Figure 21). PG models without hiPPI built at different significant pvalues show the same behavior observed when all predictors are integrated, and the scale free trend of the ki distribution is also observed when PG (without hiPPI) pvalue significance levels increases (see Figure 22). Significant PG models without hiPPI (precision ≥ 80%) also show context information of PPI in both species (see Figure 23). All these results indicate that the similarity of the

PG and KG models is not due to any bias or circular information and support all the observations, discussion and conclusions of our work.

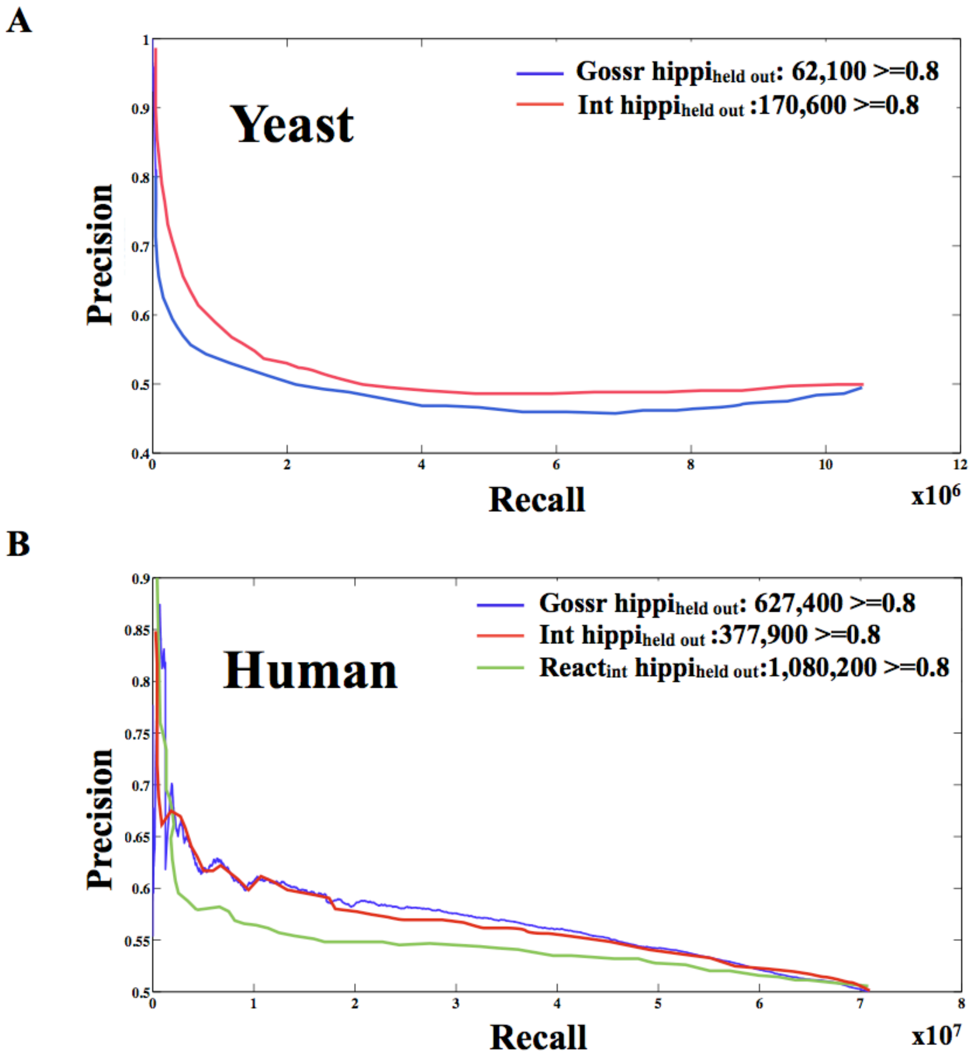

Figure 21. Prediction power of the integrated methods in yeast and human without the hiPPI predictions. Panels present the results for the yeast and human validations in terms of precision versus recall for the Fisher integration of all methods except hiPPI. In panel A validation was performed using Goss and Int datasets as gold standards in yeast. Panel B shows the validation in human using the Goss, Int and Reactome_int gold standard datasets. Number of predicted hits above 80% precision are also indicated in the panels' upper legends.

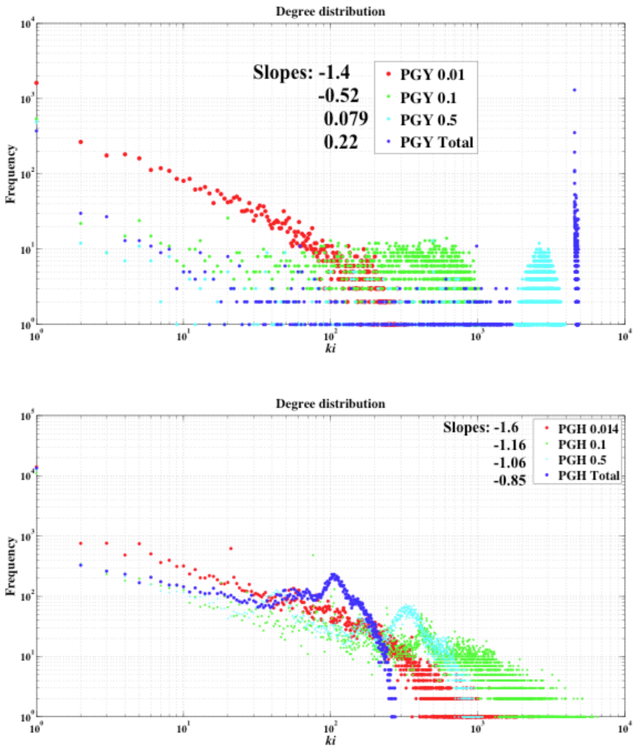

Figure 22. Degree distribution for the various PG networks built without the hiPPI predictions. Panels A and B correspond to the PG networks ki (degree) frequency distribution at different pvalue significance levels (without the hiPPI predictions) in yeast and human respectively. The legend for these panels show the exponents corresponding to the linear regression fit of the data.

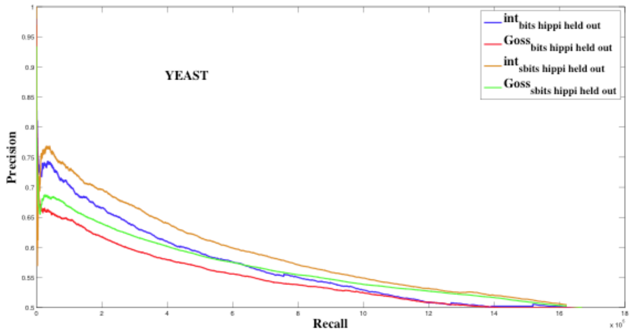

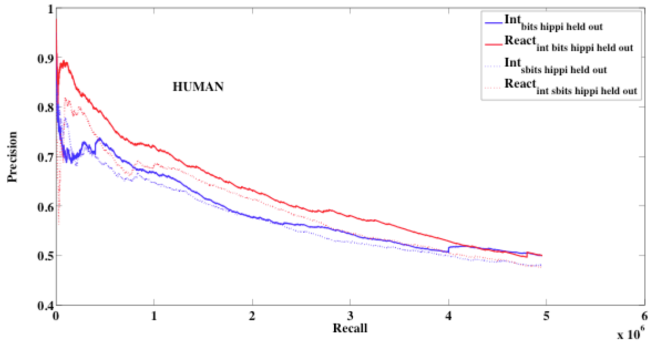

Figure 23. PG networks (without hiPPI predictions) functional context validation. These plots present the precision value (y-axis) versus specific bits similarity score (x-axis) between the interaction profiles of the protein pairs in yeast (plots A) and human (plots B) for the PG 80% precision networks without the hiPPI predictions. The gold standard dataset used, are Goss, Int in yeast and human, and Reactome_int in human.

SI references:

54. Eisen MB, Spellman PT, Brown PO, Botstein D (1998) Cluster analysis and display of genome-wide expression patterns. *Proc Natl Acad Sci* USA 95:14863-14868.
55. Grigoriev A (2001) A relationship between gene expression and protein interactions on the proteome scale: analysis of the bacteriophage T7 and the yeast Saccharomyces cerevisiae. *Nucleic Acids Res* 29:3513-3519.
56. Parkinson H, , Kapushesky M, Shojatalab M, Abeygunawardena N, Coulson R, et al. (2007) ArrayExpress--a public database of microarray experiments and gene expression profiles. *Nucleic Acids Res* 35:D747-750.
57. Enright AJ, Iliopoulos I, Kyrpides NC, Ouzounis CA (1999) Protein interaction maps for complete genomes based on gene fusion events. *Nature* 402:86-90.
58. Marcotte EM, Pellegrini M, Ng HL, Rice DW, Yeates TO, et al. (1999) Detecting protein function and protein-protein interactions from genome sequences. *Science* 285:751-753.
59. Yeats C, Lees J, Reid A, Kellam P, Martin N, Liu X, et al. (2008) Gene3D: comprehensive structural and functional annotation of genomes. *Nucleic Acids Res* 36:D414-418.

60. Watts DJ, Strogatz SH (1998) Collective dynamics of 'small-world' networks. Nature 393(6684):440-442.
61. Pruess M, Kersey P, Apweiler R (2005) The Integr8 project; a resource for genomic and proteomic data. *In Silico Biol* 5:179-185.
